# Effects of shading on morphology, photosynthesis characteristics, and yield of different shade-tolerant peanut varieties at the flowering stage

**DOI:** 10.3389/fpls.2024.1429800

**Published:** 2024-10-08

**Authors:** Jing Wang, Rui Yao, Zexin Sun, Meiwen Wang, Chunji Jiang, Xinhua Zhao, Xibo Liu, Chao Zhong, He Zhang, Shuli Zhao, Xiaoguang Wang, Haiqiu Yu

**Affiliations:** ^1^ College of Agronomy, Shenyang Agricultural University, Shenyang, China; ^2^ School of Agriculture and Horticulture, Liaoning Agricultural Vocational and Technical College, Yingkou, China

**Keywords:** peanuts, shading stress at the flowering needle stage, morphology, photosynthesis characteristics, dry-matter accumulation, yield

## Abstract

**Introduction:**

In maize and peanut intercropping, shading emerges as a critical factor for restricting peanut growth, yield, and quality.

**Methods:**

This study investigated the impact of 30% shade on shade-tolerant [Huayu 22 (HY22) and Fuhua 12 (FH12)] and shade-sensitive [Nonghua 11 (NH11) and Nonghua 5(NH5)] peanut varieties, with non-shaded condition as the control (CK). The effects of shade stress on plant morphology, photosynthetic characteristics, dry-matter accumulation, chloroplast ultra-microstructure, yield, and quality of different shade-tolerant peanut varieties were examined.

**Results:**

Compared to that in the control, shade stress led to an elongation of the main stem, shortening of the lateral branches, and reduction in the leaf area. However, these changes were less significant in the shade-tolerant than in the shade-sensitive peanut varieties, with minimal effect on the elongation of the main stem height and shortening of the lateral branches. Differences in leaf area became significant during the later stages of shade stress, particularly pronounced in the shade-sensitive peanut varieties. To enhance light capture by leaves, the shade-tolerant peanut varieties exhibited increased chlorophyll content and chloroplast grain-layer numbers. The decrease in the chlorophyll a/b ratio was more pronounced in the shade-tolerant than in the shade-sensitive peanut varieties, with significant differences. However, reduced activities of ribulose 1,5-biphosphate (RuBP) carboxylase/oxygenase and fructose 1,6-biphosphate aldolase (FBA) resulted in decreased net photosynthetic rates, particularly evident in the shade-sensitive peanut varieties during the late shade period. Shade stress led to decreased dry-matter accumulation, reduced weight of 100 fruits and kernels, and a significant decline in yield in the shade-sensitive cultivars. Shading also affected peanut-kernel quality. Compared with that in the control, the protein content increased and amino-acid (except cysteine) content decreased in the shade-tolerant cultivars.

**Discussion:**

Under shade stress, shade tolerant peanut varieties have increased the yield by improving the photosynthetic efficiency, which provided a reference for rational selection of shade tolerant peanut varieties in maize and peanut intercropping system.

## 1 Introduction

Peanut (*Arachis hypogaea* L.), originating from South America, serves as a vital source of oil and protein for humans, making it an important cash crop for both grain and oil production. It also plays a crucial role in global oil production and trade (Kong, 2014). Due to its favorable price and quantity dynamics, peanut has emerged as one of China’s agricultural products with a perennial trade surplus among oilseed crops, uniquely positioned in the international market (Chen and Lv, 2012; Fletcher and Shi, 2016). Despite China’s status as the largest peanut producer in world, domestic consumption primarily caters to meeting domestic demand. With continuous domestic-demand growth and insufficient supply, China has transitioned into a net importer of peanuts since 2019.

In recent years, with increased food demand and decreased arable land, intercropping has gained popularity as a prevalent farming model. It maximizes light energy and land-utilization rates, enhancing system stability and crop disease resistance by optimizing agro-ecosystem composition. This approach makes a sustainable ecological agriculture model, with intercropping peanuts and other tall-stalk crops as the primary planting pattern in China (Kong, 2014; Liang et al., 2021; Pelech et al., 2021). Light serves as the primary energy source for plant photosynthesis and acts as the signal for periodic plant changes (Li et al., 2017; Kaiser et al., 2019). However, intercropping imposes shade inhibition on peanut, reducing light intensity and decreasing the ratio of red to far-red light, thereby placing peanut at a long-term light disadvantage, which slows emergence and postpones flowering (Gallemí et al., 2016). The effect of shade on crops varies based on light-demand characteristics, shade period and duration, and crop-shade tolerance. Under shade conditions, plants experience reduced net photosynthetic rate, stomatal conductance, and chlorophyll content, along with damage to chloroplast ultrastructure and the photosynthetic system, ultimately leading to decreased yield (Yang et al., 2020a, b). Thus, it is essential to comprehensively investigate differential responses of various peanut varieties in morphology, photosynthesis characteristics, and yield under shading.

Shading substantially promotes the vegetative growth of crops. Chen et al. (2020) found that shading significantly increased the length of the main stem and the longest internode (the third internode from the bottom) of peanuts. Crops in intercropping exhibit a strong shade-avoidance response, with increased plant height and decreased stem diameter, branch number, aboveground biomass, pod number per plant, kernel number per plant, 100 kernel weight, and yield per plant (Cui et al., 2014; Fan et al., 2016; Chen et al., 2019a; Feng et al., 2019a). Moreover, a previous study reported decrease in the leaf-area index whereas an increase in specific leaf area due to leaf thinning (Rylski and Spigelman, 1986). Furthermore, low light intensifies the aging of the leaves, leading to a decrease in leaf number and changes in leaf size, weight, pigment, and nutrient content (Zhou et al., 2019). Wu et al. (2009) demonstrated that at seedling stage, the main stem height of peanut was significantly extended and became thinner under low light stress. In addition, dry matter accumulation, branch number per plant, leaf area and lateral branch/main stem ratio of each organ decreased, and root/shoot ratio increased, but full flowering period was delayed, flowering amount per plant significantly decreased, and pod number decreased.

Moreover, light influences photosynthesis and causes changes in the structure and function of photosynthetic organs (Li et al., 2014). Chloroplasts serve as the sites of photosynthesis (Xu et al., 2019); however, their shape, size, and quantity in plant leaves change after shade stress. After shading, the chloroplast structure in soybean leaves remains largely intact; however, some chloroplasts exhibit irregular oval shapes, with parts leaning against the cell wall, reflecting the plant’s adaptability to low-light stress, further affecting the structure and function of plant photosynthetic organs (Li et al., 2015). Additionally, changes in chlorophyll and carotenoids involved in the photosystem also affect plant photosynthetic capacity, as they are located in thylakoids grana (Demeter et al., 1974). Under shading conditions, the volume of chloroplasts and starch granules decreases, while the number of chloroplasts and the thickness of the grana layer increase. Excessive shading degree blurs the grana lamella and result in incomplete development of the chloroplast membrane and grana (Wu et al., 2014b). Furthermore, shading inhibits electron transport from photosystem II to photosystem I, reducing the electron transport rate, ATP production, and ribulose 1,5-biphosphate (RuBP) carboxylase/oxygenase activity. Additionally, photochemical quenching, quantum yield, and effective quantum yield of the photosystem are inhibited (Hussain et al., 2019a, b). Shading not only damages chloroplasts but also reduces its photosynthesis efficiency.

Dry-matter accumulation is fundamental to yield formation and exhibits a significant positive correlation with yield. However, shading causes a reduction in plant dry-matter accumulation (Gao et al., 2020). Under the substitutive strip intercropping of maize and peanut, the dry-matter accumulation in peanut was more allocated into leaves than that in stems at flowering stage for chasing more sun light (Fu et al., 2023). Also, at full seed stage, dry matter accumulation reduced by 48.1% and there was less partitioned to the pod (Fu et al., 2023). Insufficient light intensity is a critical factor; to capture more light energy, plants extend their stems, and photosynthetic products are primarily allocated to the stems. Moreover, the higher the shading degree, the greater the decrease in dry-matter accumulation, as photosynthetic capacity and product decline continuously. Underground dry-matter accumulation is more affected than aboveground dry-matter, leading to reduced root/shoot ratio and increased risk of lodging. Light significantly influences crop growth, development, yield composition, and nutritional status. Shading reduces light interception, causing a decrease in the net photosynthetic rate and carbon assimilation rate, resulting in decreases in dry weight and yield. Chen et al. (2020) observed that shade significantly reduced the number of fruits and kernels per peanut plant, resulting in a significant yield decline. Fu et al. (2023) found that the pod number of per-plant of peanut had decreased 49.9% under intercropping treatment, but also hundred-seed weight reduced significantly indicating that the quick development of maize at reproductive stage of peanut, the less pod peanut had. Wu et al. (2008) reported that when under 85% shade stress, the yield component of shade-tolerant cultivar of peanut had significantly decreased except pods per plant, but every yield component was significantly reduced in shade-sensitive cultivar, which confirmed that under shade condition, selecting shade-tolerant cultivar was of importance.

In the present study, shade-tolerant and shade-sensitive peanut varieties were analyzed to assess the shade-stress effects on plant morphology, dry-matter accumulation, photosynthesis physiology, and ultramicroscopic structure, exploring shade’s impacts on peanut yield and quality during flowering needle stage. These results will provide a theoretical reference for exploring the mechanism of peanut shade tolerance, selecting shade-tolerant peanut varieties and high-yield cultivation of intercropping.

## 2 Materials and methods

### 2.1 Material and design

This study utilized shade-tolerant peanut varieties, Huayu 22 (HY22) and Fuhua 12 (FH12), along with shade-sensitive varieties, Nonghua 11 (NH11) and Nonghua 5 (NH5), as identified in a previous study (Gao et al., 2021). All seeds were sourced from the Peanut Research Institute of Shenyang Agricultural University. The composite fertilizer used in the experiment was procured from Xinyangfeng Agricultural Technology Co., Ltd., with total nutrients ≥45% and nitrogen, phosphorus, and potassium contents of 14%, 16%, and 15%, respectively. Soil analysis of the experimental field of Shenyang Agricultural University revealed the alkali-hydrolyzed nitrogen, available phosphorus, available potassium, and organic matter contents in the 0–20 cm soil layer as 105.7 mg kg^-1^, 25.1 mg kg^-1^, 115.3 mg kg^-1^, and 15.37 g kg^-1^, respectively.

The experiment was conducted at the agricultural and rural department of Northeast Region Crop Cultivation Scientific Observation Experiment Station of Shenyang Agricultural University in 2020 and 2021 (E123°25’31.18’’, N41°48’ 11.75’’). It involved two treatments: 30% shade and no shade (control) (Rao and Zhao, 1989; Peng and Chen, 1999; Li et al., 2009). The field experiment was arranged in randomized blocks, with each treatment repeated three times. Each plot consisted of five rows oriented along a north-south ridge to get more solar energy, measuring 7 m in length, with intra row spacing of 0.580 m. Each hectare had 150,000 holes with 0.115 m, and two seeds planted per hole. The shade net was used for shade treatment around July 7^th^ at the flowering needle stage namely after 55 days after planting, using a shade shed with a height of 1.5 m. The net was positioned 30-cm above the ground on the east, south, and west sides, while the north side remained completely open for ventilation. Photosynthetic active radiation was measured using an AccuPAR LP-80 (METER Group Inc, Pullman, Washington, USA) at 9:00 am, 11:00 am, 1:00 pm, and 3:00 pm on sunny days, 2 m from the ceiling of the shade net. The shade net had a 30% shading rate, with an actual shading rate of 28.7%. Samples were collected at 10, 20, 30, 40, and 50 days after shading.

### 2.2 Collection of shade related indices

#### 2.2.1 Morphological characteristics

Five representative plants were selected from each plot to determine main stem height and lateral-branch length, and leaf area using a ruler and the fresh-sample punching-and-weighing method, respectively.

#### 2.2.2 Determination of dry matter dynamic accumulation

The plants were cleaned, and peanut organs were separated, placed in kraft paper bags, and oven-dried at 105°C for 30 min to inactive enzymes or bacterial and fungus, followed by drying at 85°C to make dry matter characteristics unchanged until reaching a constant weight. The dried samples were weighed and recorded (Gao et al., 2024).

#### 2.2.3 Determination of photosynthetic characteristics

##### 2.2.3.1 Chlorophyll content

Leaf samples were cut away from the veins and weighed (approximately 0.1 g), soaked in 95% ethanol, and kept in dark for 48 h. The light absorption values of the extracted solution were measured using an ultraviolet spectrophotometer at wavelengths of 665, 649, and 470 nm (Xu et al., 2024). The chlorophyll content was calculated using the following formula:


$$Chlorophyll\;a\left(mg\cdot L^{-1}\right)=13.95\cdot D_{665}-6.88\cdot D_{649}$$

$$Chlorophyll\;b\left(mg\cdot L^{-1}\right)=24.96\cdot D_{649}-7.32\cdot D_{665}$$

$$Chlorophyll\;a+b\left(mg\cdot g^{-1}\right)=\left(Ca\;+\;Cb\right)\times V\times n/m$$

$$Chlorophyll\;a/b=C_{a}/C_{b}$$

Here, D665, D649, and D470 represent the absorbance of chloroplast pigment extracts at wavelengths 665, 649, and 470 nm, respectively. C - pigment concentration (mg·L^-1^); V - the volume of the extract (L); n - dilution ratio; m - fresh leaf weight (g).

##### 2.2.3.2 Photosynthetic parameter

The portable CIRAS-2 photosynthmeter (Hansatech Instruments Ltd., King’s Lynn, Norfolk, UK) was used to determine Pn, Gs, Ci, and Tr of leaves with normal growth of the main stem on sunny days from 9:00 to 11:00 am every 10 days after shading (Wang et al., 2023). All treatments were measured at least three times.

##### 2.2.3.3 Photosynthetic-related enzyme activity

Fresh peanut leaves (0.5 g) from the penultimate main stem were collected every 10 days after shading, frozen with liquid nitrogen, and stored in a refrigerator at -80°C. The activities of RuBP carboxylase/Oxygenase and FBA in leaves were determined using the Rubisco-bisphosphate Carboxylase/Oxygenase Activity Assay Kit (BC0445, Solarbio, Beijing Solarbio Science and Technology Co., Ltd., Beijing, China) and FBA Activity Assay Kit (BC2275, Solarbio, Beijing Solarbio Science and Technology Co., Ltd), respectively, using enzyme-linked immunoassay (Li et al., 2017; Bai et al., 2024). For Rubisco-bisphosphate Carboxylase/Oxygenase Activity and FBA Activity determination, 0.1 g leaves was collected, added 1 mL extraction solution and ground into homogenate, centrifuged according to instruction, collected supernatant and then centrifuged (SCI-NTZ-48, Zhuhai Heima instrument company, China).

Absorbance (OD value) was measured at 340 nm with an ELIASA (Thermo Scientific Multiskan GO, Thermo Fisher Scientific Oy Ratastie 2, FINLAND). The RuBP carboxylase (U·L^-1^) and FBA activities (U·L^-1^) were calculated according to the standard curve equation.

##### 2.2.3.4 Observation of the chloroplast ultrastructure

After 50 days of shade, leaf segments measuring 1 mm × 1 mm × ~2–3 mm on both sides of the main vein were excised. These segments were immediately placed into a penicillin bottle with fixation solution, washed with phosphoric acid buffer, then dehydrated, embedded, sliced, and stained in sequence. The ultrastructure of the leaves was observed under a JEM-1200EX (JEOL CO., LTD, Mitaka City, Tokyo, Japan) transmission electron microscope (Luo et al., 2021).

#### 2.2.4 Yield, yield component, and kernel quality analysis

Upon harvest, 10 plants were selected from a representative and complete row in each plot, and the entire plant was placed into a gauze bag and dried until reaching a constant weight. The following parameters were determined: number of fruit branches, number of fruit per plant, number of full fruit per plant, fruit weight per plant, 100-fruit weight, and 100-kernel weight. From each plot, three complete rows from the middle section were selected. The ends of each row were discarded (0.5 m), and the total number of plants was counted. Pods were harvested and placed into gauze bags, dried to a constant weight, and then used to calculate the yield (Gao et al., 2024).

The percentage of fat, protein, fatty acid, and amino acid components in peanut kernel were determined using a FOSS near-infrared grain analyzer (Infratec TM NOVA, Denmark).

### 2.3 Statistical analysis

Statistical analysis was performed using Microsoft Excel 2019 and DPSv7.05 and ANOVA (*P*<0.05; *P*<0.01) was used for data analysis. Microsoft Excel 2019 and GraphPad Prism 8 software were employed to generate charts.

## 3 Results

### 3.1 Effect of shade on the morphology of peanut at the flowering needle stage

**Figure 1:**
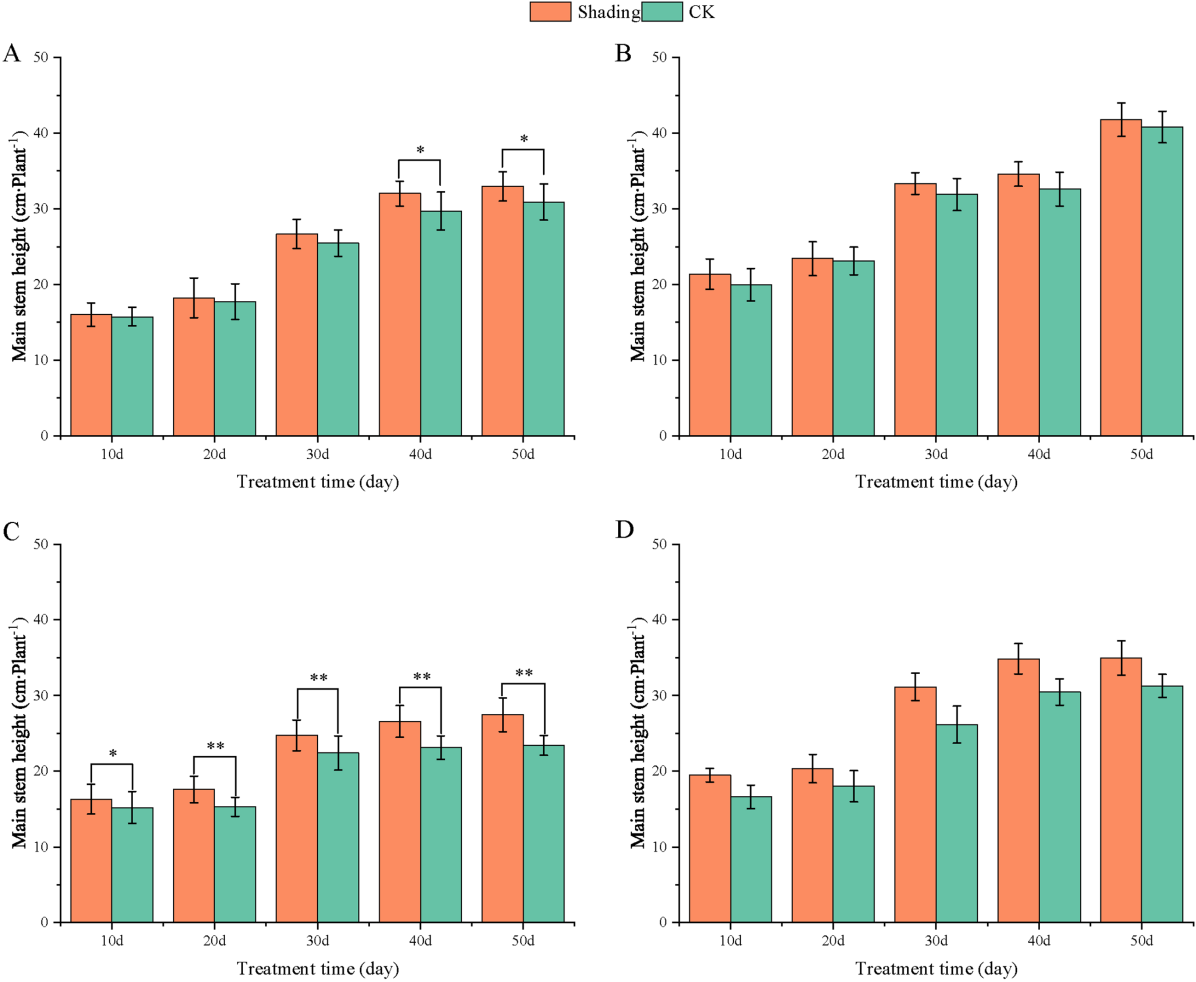
Effect of shading on the main stem height of peanut at the flowering needle stage (2020). **(A)**, HY22; **(B)**, FH12; **(C)**, NH11; **(D)**, NH5. Y-axis represents main stem height, x-axis represents the days of different cultivars under shade stress. Values are mean of three replicates, bars indicate ± standard error, and stars indicate significant differences between shade and full sun (*P<0.05, **P<0.01).

**Figure 2:**
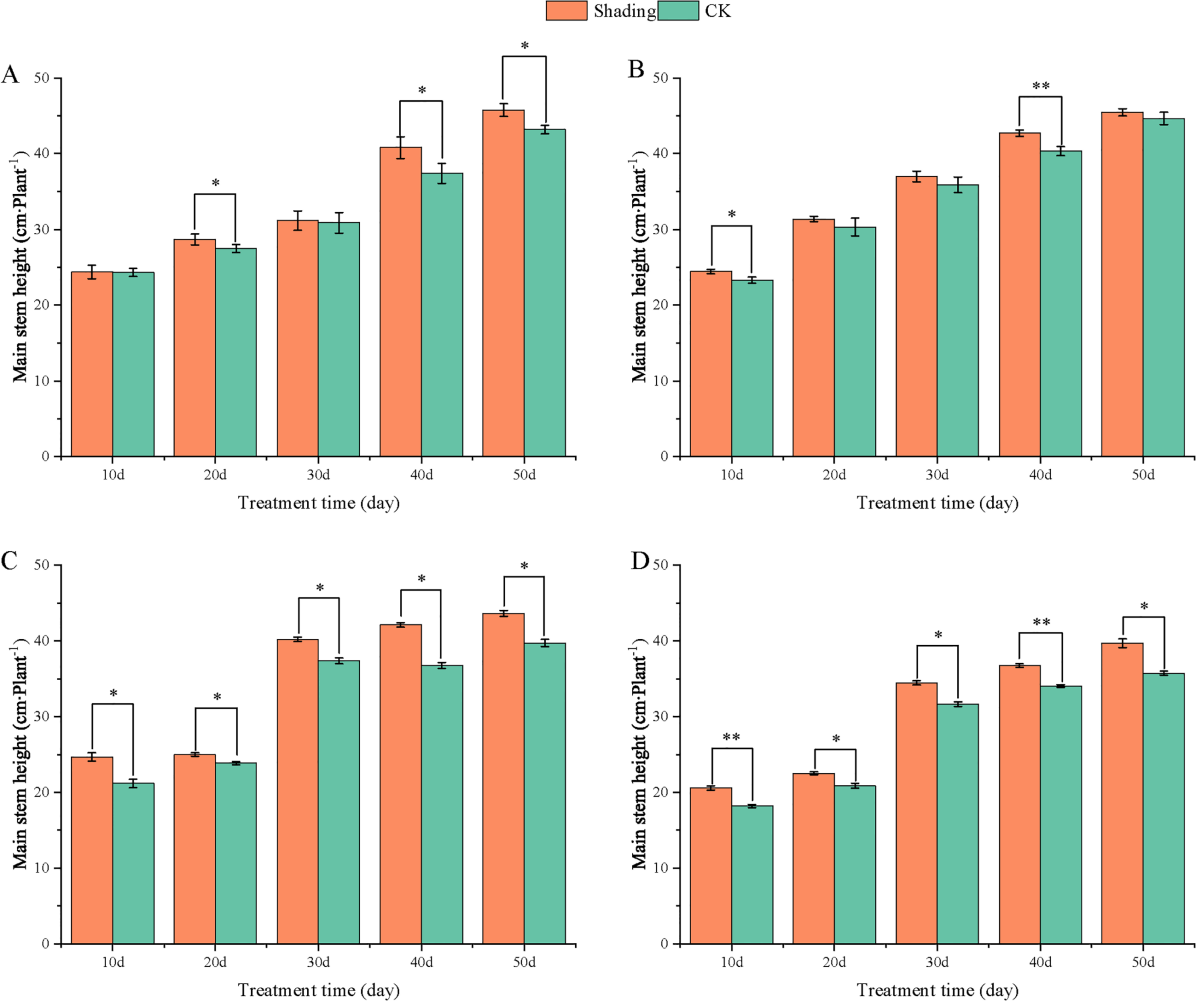
Effect of shading on the main stem height of peanut at the flowering needle stage (2021). **(A)**, HY22; **(B)**, FH12; **(C)**, NH11; **(D)**, NH5. Y-axis represents main stem height, x-axis represents the days of different cultivars under shade stress. Values are mean of three replicates, bars indicate ± standard error, and stars indicate significant differences between shade and full sun (*P<0.05, **P<0.01).

**Figure 3:**
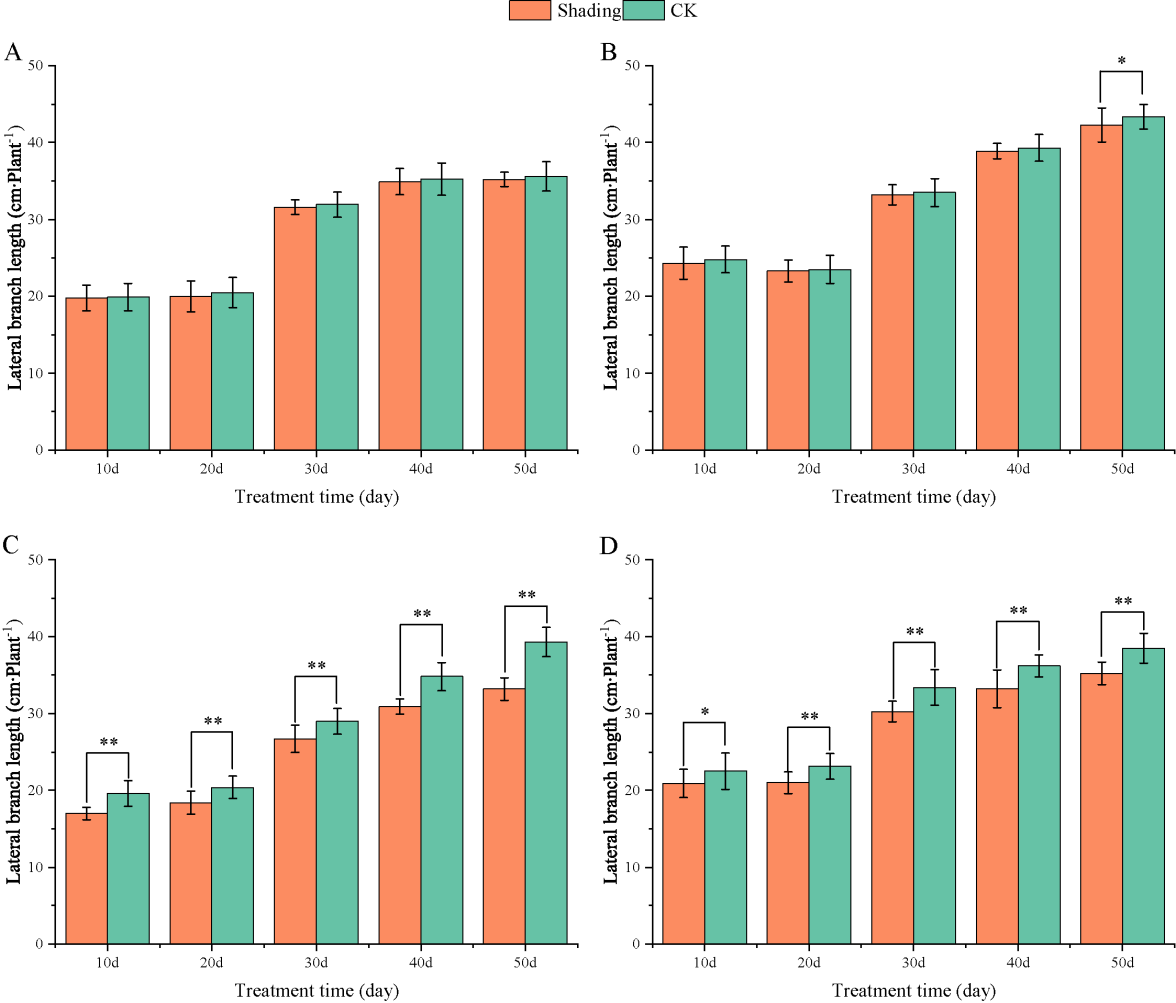
Effect of shading on lateral branch length of peanut at the flowering needle stage (2020). **(A)**, HY22; **(B)**, FH12; **(C)**, NH11; **(D)**, NH5. Y-axis represents lateral branch length, x-axis represents the days of different cultivars under shade stress. Values are mean of three replicates, bars indicate ± standard error, and stars indicate significant differences between shade and full sun (*P<0.05, **P<0.01).

**Figure 4:**
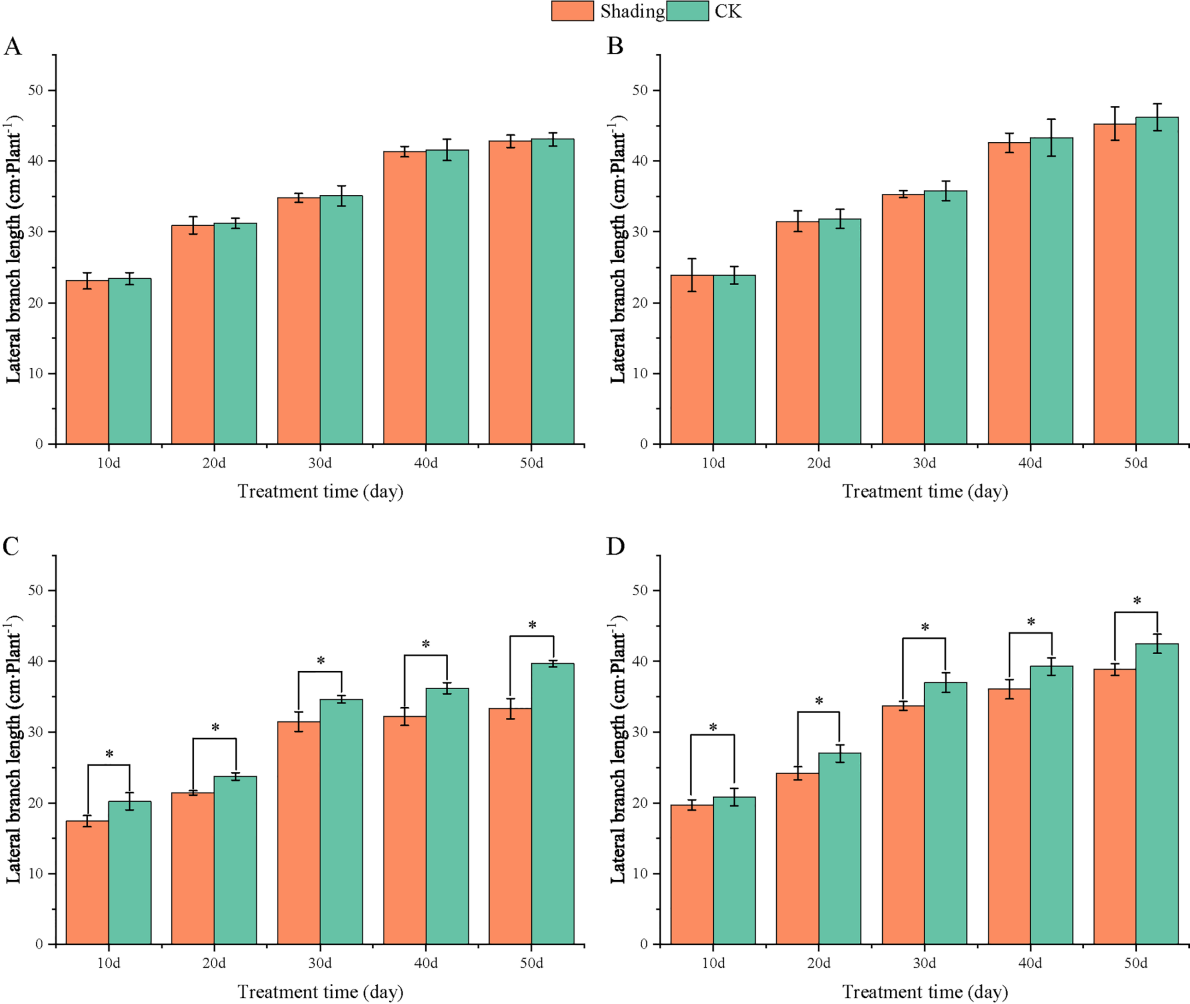
Effect of shading on lateral branch length of peanut at the flowering needle stage (2021). **(A)**, HY22; **(B)**, FH12; **(C)**, NH11; **(D)**, NH5. Y-axis represents lateral branch length, x-axis represents the days of different cultivars under shade stress. Values are mean of three replicates, bars indicate ± standard error, and stars indicate significant differences between shade and full sun (*P<0.05).

**Figure 5:**
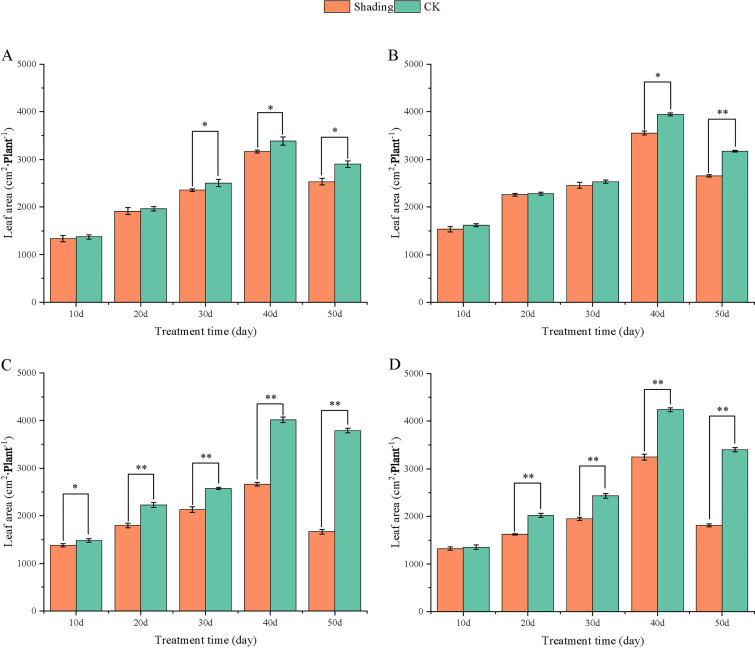
Effect of shading on leaf area of peanut at the flowering needle stage (2020). **(A)**, HY22; **(B)**, FH12; **(C)**, NH11; **(D)**, NH5. Y-axis represents leaf area, x-axis represents the days of different cultivars under shade stress. Values are mean of three replicates, bars indicate ± standard error, and stars indicate significant differences between shade and full sun (*P<0.05, **P<0.01).

**Figure 6:**
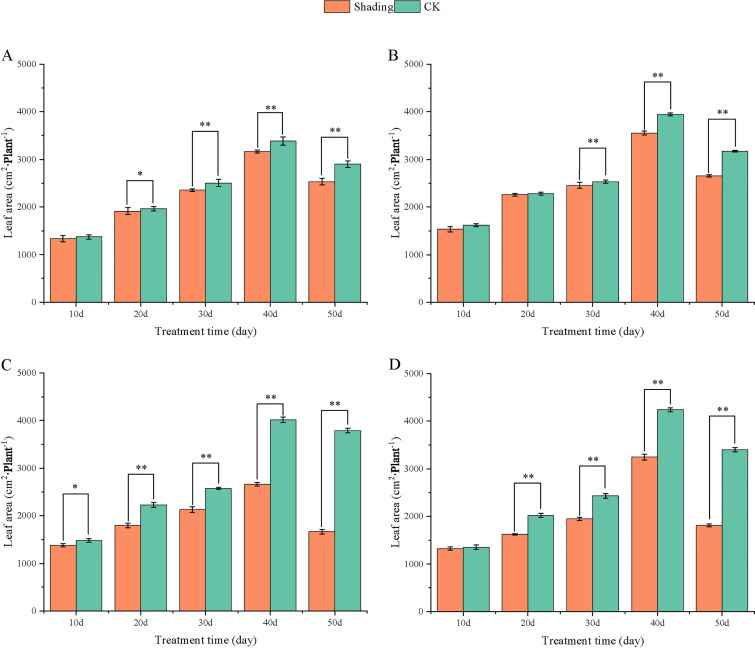
Effect of shading on leaf area of peanut at the flowering needle stage (2021). **(A)**, HY22; **(B)**, FH12; **(C)**, NH11; **(D)**, NH5. Y-axis represents leaf area, x-axis represents the days of different cultivars under shade stress. Values are mean of three replicates, bars indicate ± standard error, and stars indicate significant differences between shade and full sun (*P<0.05, **P<0.01).

To investigate the effect of shading stress on the height of peanut main stems, measurements were taken every 10 days after shading. Compared to the control group, both shade-tolerant peanut varieties exhibited a gradual increase in main-stem height after shading (**Figures 1**, **2**). The main-stem height and the stem heights of the shade-tolerant peanut cultivar HY22 in 2020 and 2021 were 7.85%and 6.77%, and 9.09%, 5.95% (*P<* 0.05) higher than that of the control at 40 days and 50 days after shade treatment, respectively; similar results were also found in the shade-tolerant cultivar FH12. In both 2020 and 2021, the maximum growth of NH11 occurred at 50 and 10 days after shading, 17.31% and 13.19% higher than that of the control, respectively. For NH5, maximum growth occurred at 10 (17.47%) and 10 (16.35%) days after shading, respectively. These results indicated that shade stress promoted the elongation and growth of the main stem of peanut varieties, particularly in shade-sensitive varieties. Compared with that of the control group, the lateral branch length of different shade-tolerant peanut varieties gradually decreased after shade stress during the flower needle stage (**Figures 3**, **4**). While the differences were not significant, the lateral branch length of HY22 in 2020 and 2021 was 0.05%–2.44% and 0.58%–1.14% shorter than that of the control, respectively, at 10–50 days after shading. Similarly, FH12 showed reductions of 0.81%–2.47% and 0.01%–1.95%, respectively, which were significant at 50 d. The lateral branch lengths of NH11 were reduced by 13.3%, 9.8%, 7.77%, 11.2%, 15.52%, and 13.8%, 9.78%, 9.09%, 11.05%, 16.12%, compared with that of the control 10–50 days after shading in 2020 and 2021, respectively, and the differences were highly significant and significant; while in NH5, it decreased by 7.2%, 9.09%, 9.43%, 8.28%, 8.57% and 5.5%, 10.31%, 8.85%, 8.10%, 8.43%. These results indicated that the lateral branches of peanut were shortened by shade stress, particularly in shade-sensitive peanut varieties, resulting in incomplete development. Similarly, the leaf area of different shade-tolerant peanut varieties showed a trend of gradual decrease after shade stress (**Figures 5**, **6**). In 2020, the leaf areas of the shade-tolerant peanut varieties HY22 and FH12 were not significantly different from 10 to 30 days after shading; however, reductions of 9.69% and 14.54% and 12.37% and 11.84% were observed after shading, respectively, with highly significant differences. In 2021, similar trends were observed, the decrease were highly significant at 30-50 days. The leaf area of shade-sensitive peanut varieties NH11 and NH5 was highly significant different from that of the control at 20 days after shading, decreasing by 27.91%–50.53% and 23.44%–36.35% from 20 to 50 days, respectively, after shading in 2020. In 2021, the leaf area was reduced by 19.24%–56.08% and 19.75%–46.73%, respectively, with highly significant differences.

### 3.2 Effects of shade on dry-matter accumulation in the organs of peanut at the flowering needle stage

**Figure 7:**
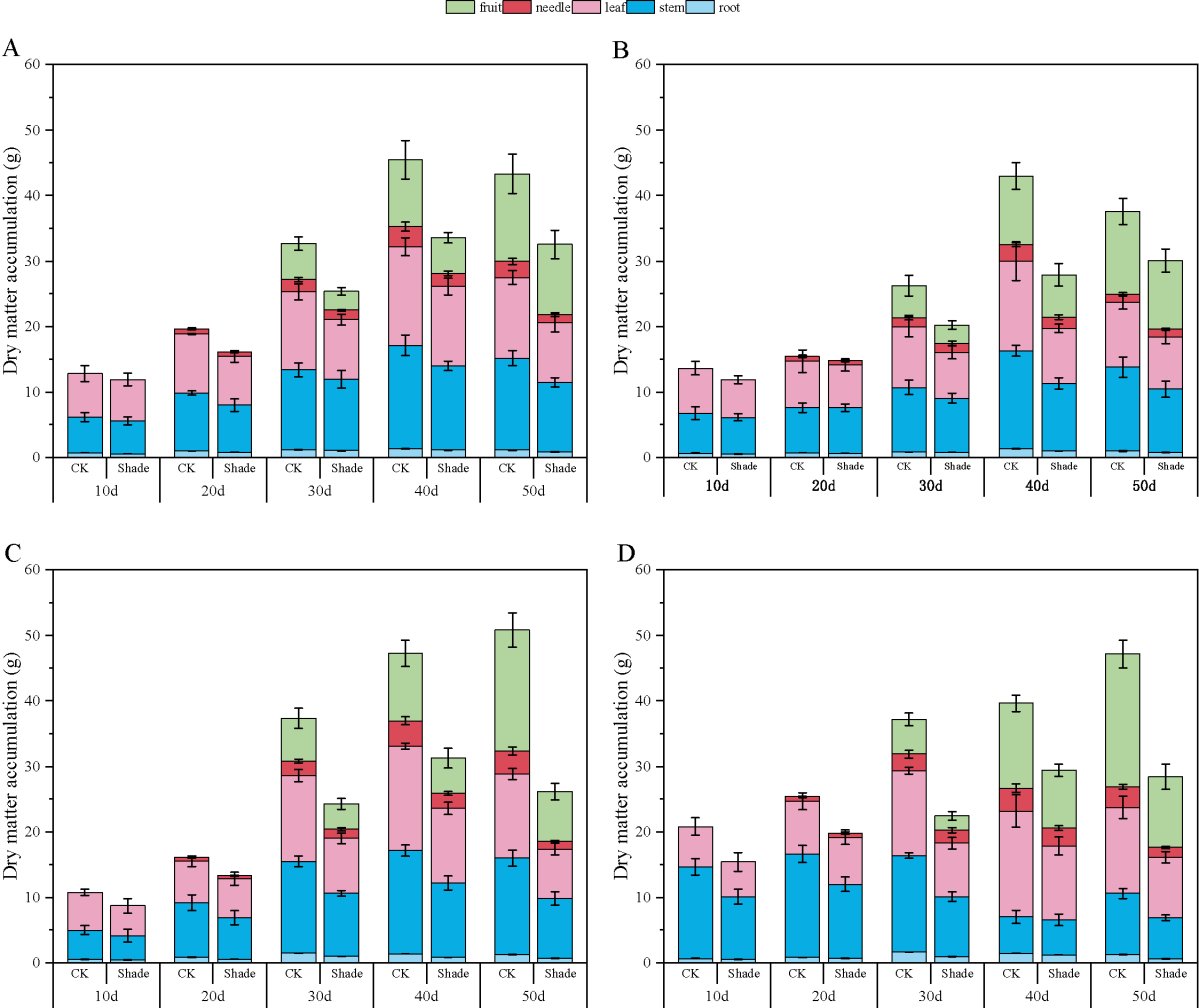
Effect of shading on dry-matter accumulation of peanut at the flowering needle stage (2020). **(A)**, HY22; **(B)**, FH12; **(C)**, NH11; **(D)**, NH5. Y-axis represents dry-matter accumulation, x-axis represents the days of different cultivars under shade stress. Values are mean of three replicates, bars indicate ± standard error. Green bar indicates fruit, red bar indicates needle, pink bar indicates leaf, blue bar indicates stem, light blue indicates root.

**Figure 8:**
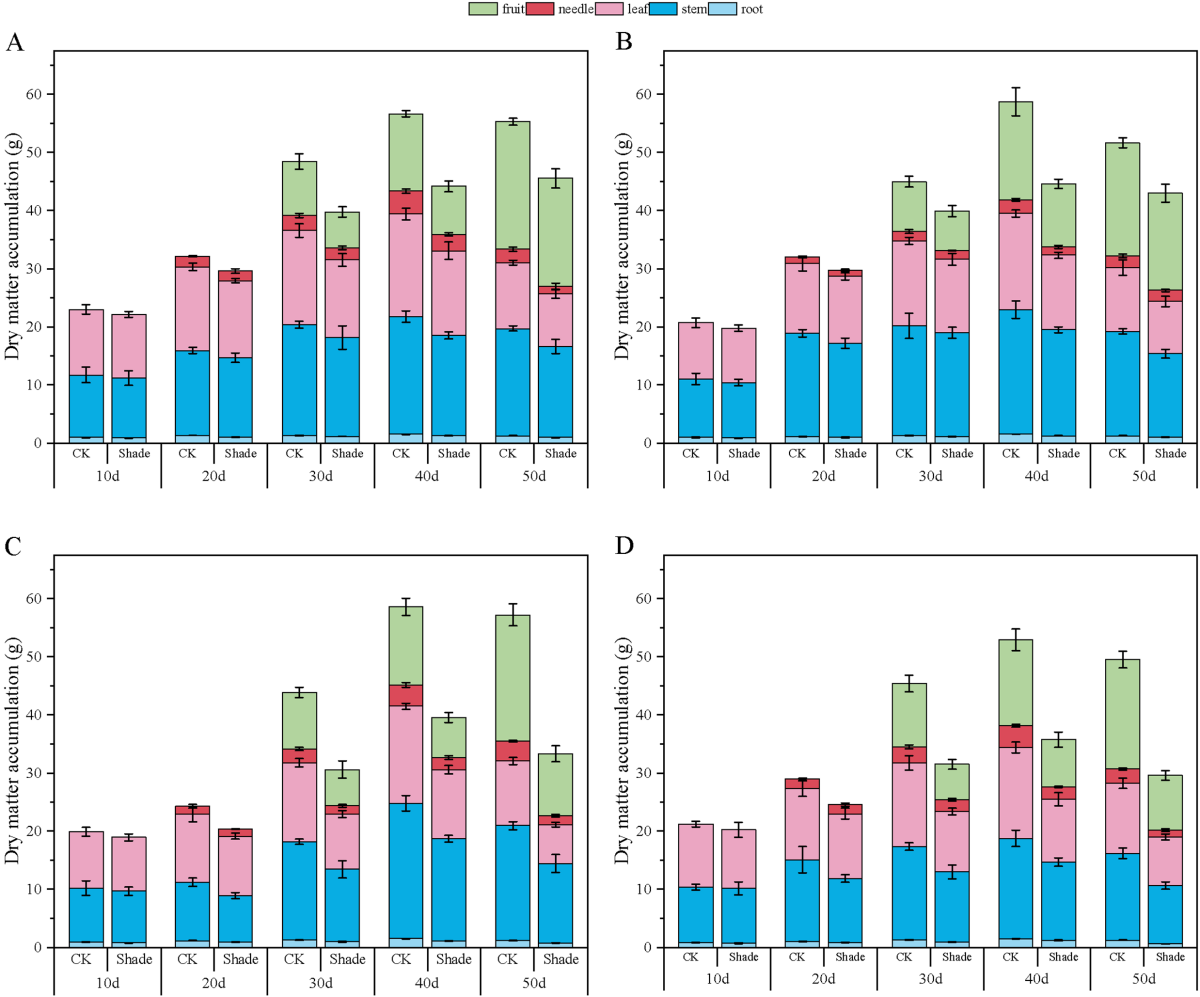
Effect of shading on dry-matter accumulation of peanut at the flowering needle stage (2021). **(A)**, HY22; **(B)**, FH12; **(C)**, NH11; **(D)**, NH5. Y-axis represents dry-matter accumulation, x-axis represents the days of different cultivars under shade stress. Values are mean of three replicates, bars indicate ± standard error. Green bar indicates fruit, red bar indicates needle, pink bar indicates leaf, blue bar indicates stem, light blue indicates root.

The dry-matter accumulation in peanuts initially increased and then slightly decreased as the growth progressed (**Figures 7**, **8**). Toward the later growth stages, some leaves and fruit needles decayed, resulting in a slight decrease in the overall dry-matter accumulation. Herein, the accumulation of dry matter in the organs of different shade-tolerant peanut varieties decreased.

In 2020, the root dry-matter accumulation of shade-tolerant peanut varieties HY22 and FH12 showed no significant difference compared to that of the control at the early stage of shading but decreased by 16.30% and 22.76%, and 26.70% and 23.99% at 40 and 50 days after shading, respectively, with significant differences (*P*< 0.05). NH11 and NH5 decreased by 17.19%–45.92% and 16.42–50.87% 10–50 days after shading, respectively, with significant differences (*P*< 0.05) (**Figure 7**). Similar results were obtained in 2021 (**Figure 8**). Compared to that by the control, the shade-tolerant peanut varieties exhibited no significant difference in the first 30 days after shading, reaching a significant difference level at 40 and 50 days after shading (*P*< 0.05), while the shade-sensitive peanut varieties exhibited a significantly huge decrease at 10–50 days after shading (*P*< 0.05). In 2020 and 2021, the stem dry-matter accumulation of the shade-tolerant peanut varieties HY22 and FH12 exhibited no significant difference from that of the control group at 10–40 days after shading; however, the stem dry-matter accumulation decreased by 24.20% and 24.50% and 15.00% and 20.17%, respectively with significant differences (*P*< 0.05) at 50 days after shading compared with that in the control. The stem dry-matter accumulation of NH11 and NH5 varieties decreased by 28.15%–37.73% and 32.75%–37.73% and 24.53%–30.68% and 22.00%–32.81%, respectively, after 30–50 days of shading, with significant differences (*P*< 0.05) (**Figures 7**, **8**). In 2020, the dry-matter accumulation in leaves of the shade-tolerant peanut varieties HY22 and FH12 decreased by 19.65% and 26.30% and 19.49% and 38.42% compared with that in the control at 30 and 40 days after shade, and the differences were significant (*P*< 0.05) (**Figure 7**). In 2021, the dry-matter accumulation of the shade-tolerant peanut varieties HY22 and FH12 decreased by 17.56% and 22.52%, respectively, 40 days after shading, with significant differences (*P*< 0.05). In 2020 and 2021, the dry matter accumulation the shade-sensitive peanut varieties NH11 and NH5 decreased by 28.57%–41.43% and 29.57%–37.04%, and 28.83%–40.26% 27.95%–31.10%, respectively, with significant difference (*P*< 0.05) after 30–50 days of shading (**Figure 8**).In 2020 and 2021, the shade-tolerant varieties HY22 and FH12 showed no significant difference in dry-matter accumulation of fruit needles between 20 and 50 days after shade, while the shade-sensitive varieties NH11 and NH5 showed no significant difference between 20 and 40 days after shade. At 50 days, dry-matter accumulation decreased by 66.91% and 53.66% and 55.12% and 52.98%, respectively, compared with that in the control group, with significant differences (*P*< 0.05) (**Figures 7**, **8**). In 2020, the pod dry-matter accumulation of HY22 and FH12 decreased by 47.48% and 46.48% and 42.01% and 48.08% compared with that in the control at 30 and 40 days after shade, respectively, and the differences were significant (*P*< 0.05). However, no significant difference was observed at 50 days after shading compared to that in the control. NH11 and NH5 were significantly reduced by 40.63%–58.72% and 31.54%–59.17% after 30–50 days of shading, respectively, compared to that in the control (*P*< 0.05) (**Figure 7**), with similar results in 2021.The total dry matter of HY22 and FH12 in 2020 was not significantly different at 10–30 days after shade; however, it decreased by 26.18% and 24.92% and 35.15% and 19.97% (*P*< 0.05) at 40 and 50 days after shade, respectively. The total dry matter of NH11 and NH5 had no significant difference at 10 and 20 days but decreased by 33.81%–48.51% and 25.77%–39.74% (*P*< 0.05) at 30–50 days after shade, respectively. The results in 2021 were similar, with no significant difference between the shade-intolerant peanut varieties at 10–30 days after shading, and significant difference observed at 40 and 50 days after shading (*P*< 0.05); however, the shade-sensitive peanut varieties exhibited significant difference at 30–50 days after shade (*P*< 0.05) (**Figure 8**). These results indicate that the dry-matter accumulation of the shade-tolerant peanut varieties was greater than that of the shade-sensitive peanut varieties under shade stress at the flowering stage.

### 3.3 Effect of shade on chloroplast pigment content of peanut at the flowering needle stage

Compared with that in the control, the chlorophyll a content in the leaves of different shade-tolerant peanut varieties showed an increasing trend under shade stress at the flowering stage. It increased by 6.87%–26.78% and 10.16%–23.44% from 10 to 50 days after shade stress, respectively, with significant differences (*P*< 0.05) in HY22 and FH12 (**Table 1**). However, there were no significant differences at 10 and 20 days after shade but was 8.49%–14.18% and 11.67%–12.32% higher than the control at 30–50 days, respectively, with significant differences (*P*< 0.05) in NH11 and NH5. At 30 days after shading, the increase rate of shade-tolerant peanut varieties was the highest at 26.78% and 23.44%, respectively, for the shade-tolerant peanut varieties, and 14.18% and 12.32%, respectively, for the shade-sensitive varieties, and both differences were significant (*P<* 0.05).

**Table 1:** Effect of shading on chlorophyll a content in peanut leaves at the flowering needle stage (2021).

Cultivar	Treatment	Treatment days				
		10	20	30	40	50
HY22	CK	1.55 ± 0.02a	1.69 ± 0.01a	1.25 ± 0.03a	1.10 ± 0.01a	1.06 ± 0.02a
	Shade	1.73 ± 0.01b	1.81 ± 0.01b	1.59 ± 0.01b	1.32 ± 0.01b	1.22 ± 0.02b
FH12	CK	1.49 ± 0.01a	1.62 ± 0.02a	1.28 ± 0.02a	1.12 ± 0.01a	1.08 ± 0.01a
	Shade	1.69 ± 0.02b	1.78 ± 0.01b	1.58 ± 0.03b	1.37 ± 0.02b	1.23 ± 0.02b
NH11	CK	1.71 ± 0.02a	1.77 ± 0.02a	1.42 ± 0.01a	1.31 ± 0.01a	1.28 ± 0.01a
	Shade	1.83 ± 0.02a	1.86 ± 0.03a	1.62 ± 0.03b	1.44 ± 0.01b	1.39 ± 0.02b
NH5	CK	1.57 ± 0.03a	1.82 ± 0.04a	1.38 ± 0.02a	1.16 ± 0.01a	1.10 ± 0.01a
	Shade	1.71 ± 0.02b	1.91 ± 0.02a	1.55 ± 0.02b	1.31 ± 0.02b	1.23 ± 0.01b

Different lowercase letters in the same column in each index indicated significant differences among samples (P<0.05), ± indicates standard error.

Unit: mg·g^-1^

Chlorophyll b absorbs light energy and transfers it to chlorophyll a, and its content directly affects photosynthesis. Chlorophyll b content in the leaves of different shade-tolerant peanut varieties also increased under shade stress at the flowering stage. It increased by 18.23%–46.34% and 24.00%–49.75% compared with that in the control at 10–50 days after shading, respectively, with significant differences (*P*< 0.05) in HY22 and FH12 (**Table 2**). It increased by 13.38%, 23.49%, and 15.86% at 10, 30, and 50 days after shading, respectively, compared to that the control, with significant differences (*P*< 0.05). However, no significant difference was observed in NH11 at 20 and 40 days after shading (**Table 2**). Similar results were observed in NH5, with a significant difference at 40 days after shading but not at 50 days.

**Table 2:** Effect of shading chlorophyll b content in peanut leaves at the flowering needle stage on (2021).

Cultivar	Treatment	Treatment days				
		10	20	30	40	50
HY22	CK	0.43 ± 0.02b	0.54 ± 0.01b	0.36 ± 0.01b	0.34 ± 0.01b	0.31 ± 0.01b
	Shade	0.58 ± 0.01a	0.64 ± 0.01a	0.52 ± 0.01a	0.46 ± 0.01a	0.42 ± 0.01a
FH12	CK	0.44 ± 0.02b	0.50 ± 0.02b	0.35 ± 0.01b	0.33 ± 0.01b	0.30 ± 0.01b
	Shade	0.58 ± 0.01a	0.62 ± 0.01a	0.53 ± 0.01a	0.48 ± 0.01a	0.42 ± 0.01a
NH11	CK	0.49 ± 0.02a	0.52 ± 0.02a	0.40 ± 0.01a	0.39 ± 0.01a	0.36 ± 0.01a
	Shade	0.56 ± 0.02b	0.58 ± 0.01a	0.49 ± 0.01b	0.45 ± 0.01a	0.41 ± 0.01b
NH5	CK	0.40 ± 0.02b	0.55 ± 0.03a	0.39 ± 0.01b	0.34 ± 0.01b	0.32 ± 0.02a
	Shade	0.48 ± 0.02a	0.60 ± 0.05a	0.47 ± 0.01a	0.42 ± 0.01a	0.37 ± 0.01a

Different lowercase letters in the same column in each index indicated significant differences among samples (P<0.05), ± indicates standard error.

Unit: mg·g^-1^

Chlorophyll a+b content in peanut leaves initially increased and then decreased with plant growth. Compared to that in the control, the chlorophyll a+b contents of different shade-tolerant peanut varieties increased under shade stress at the flowering stage. The values rose by 9.63%–31.10% and 13.43%–29.14% compared with that of the control at 10–50 days after shade, respectively, and the differences were significant (*P*< 0.05) (**Table 3**). The chlorophyll a+b contents in NH11 and NH5 showed no significant differences at 10 and 20 days after shading; however, they increased by 10.09%–16.23% and 12.39%–14.47% compared with that in the control at 30–50 days after shading, respectively, and the differences were significant (*P*< 0.05) (**Table 3**). These results indicate that shade stress promoted chlorophyll synthesis, with greater increases observed in shade-tolerant peanut varieties.

**Table 3:** Effect of shading on chlorophyll a+b content in peanut leaves at the flowering needle stage (2021).

Cultivar	Treatment	Treatment days				
		10	20	30	40	50
HY22	CK	1.99 ± 0.05b	2.23 ± 0.06b	1.61 ± 0.02b	1.44 ± 0.04b	1.37 ± 0.01b
	Shade	2.31 ± 0.02a	2.45 ± 0.05a	2.11 ± 0.01a	1.78 ± 0.03a	1.64 ± 0.01a
FH12	CK	1.93 ± 0.03b	2.12 ± 0.02b	1.63 ± 0.02b	1.45 ± 0.01b	1.38 ± 0.03b
	Shade	2.27 ± 0.05a	2.40 ± 0.02a	2.11 ± 0.03a	1.85 ± 0.02a	1.65 ± 0.02a
NH11	CK	2.20 ± 0.06a	2.29 ± 0.08a	1.82 ± 0.01b	1.70 ± 0.02b	1.64 ± 0.02b
	Shade	2.38 ± 0.01a	2.44 ± 0.05a	2.11 ± 0.01a	1.89 ± 0.01a	1.80 ± 0.01a
NH5	CK	1.97 ± 0.04a	2.37 ± 0.03a	1.77 ± 0.02b	1.51 ± 0.02b	1.42 ± 0.01b
	Shade	2.19 ± 0.07a	2.51 ± 0.04a	2.02 ± 0.01a	1.72 ± 0.02a	1.60 ± 0.01a

Different lowercase letters in the same column in each index indicated significant differences among samples (P<0.05), ± indicates standard error.

Unit: mg·g^-1^

Chlorophyll a+b plays an important role in photosynthetic electron transport, and a higher ratio is conducive to improving the ability to capture weak light. Leaf chlorophyll a/b of different shade-tolerant peanut varieties showed a downward trend after shade stress at flowering stage. It decreased in HY22 and FH12 by 9.60%–16.50% and 11.16%–18.80% at 10–50 days of shade stress, respectively, and the differences were significant (*P*< 0.05) (**Table 4**). In contrast, it decreased by 5.60% and 9.35%, respectively, at 10 days after shade stress compared to that in the control, with the differences reaching significant levels (*P*< 0.05) (**Table 4**); however, there was no significant difference at 20–50 days after shade stress in NH11 and NH5, but for 30 days in NH5. Under shade stress, chlorophyll a+b content of the shade-tolerant peanut varieties decreased significantly, indicating a larger proportion of chlorophyll b, which was conducive to capture short-wave radiation and improve light energy utilization.

**Table 4:** Effect of shading on chlorophyll a/b of peanut at the flowering needle stage (2021).

Cultivar	Treatment	Treatment days				
		10	20	30	40	50
HY22	CK	3.57 ± 0.03a	3.13 ± 0.03a	3.52 ± 0.02a	3.24 ± 0.06a	3.41 ± 0.07a
	Shade	2.98 ± 0.04b	2.83 ± 0.03b	3.05 ± 0.06b	2.87 ± 0.01b	2.90 ± 0.04b
FH12	CK	3.39 ± 0.04a	3.24 ± 0.02a	3.62 ± 0.05a	3.39 ± 0.09a	3.61 ± 0.03a
	Shade	2.91 ± 0.01b	2.88 ± 0.04b	2.98 ± 0.02b	2.84 ± 0.05b	2.93 ± 0.03b
NH11	CK	3.48 ± 0.04a	3.40 ± 0.04a	3.54 ± 0.08a	3.36 ± 0.04a	3.60 ± 0.04a
	Shade	3.29 ± 0.01b	3.21 ± 0.17a	3.28 ± 0.05a	3.21 ± 0.08a	3.37 ± 0.06a
NH5	CK	3.92 ± 0.01a	3.33 ± 0.02a	3.56 ± 0.05a	3.41 ± 0.04a	3.41 ± 0.05a
	Shade	3.55 ± 0.06b	3.19 ± 0.05a	3.30 ± 0.04b	3.12 ± 0.06a	3.32 ± 0.05a

Different lowercase letters in the same column in each index indicated significant differences among samples (P<0.05), ± indicates standard error.

### 3.4 Effect of shade on photosynthesis of peanut at the flowering needle stage

#### 3.4.1 Photosynthetic physiological parameters

**Figure 9:**
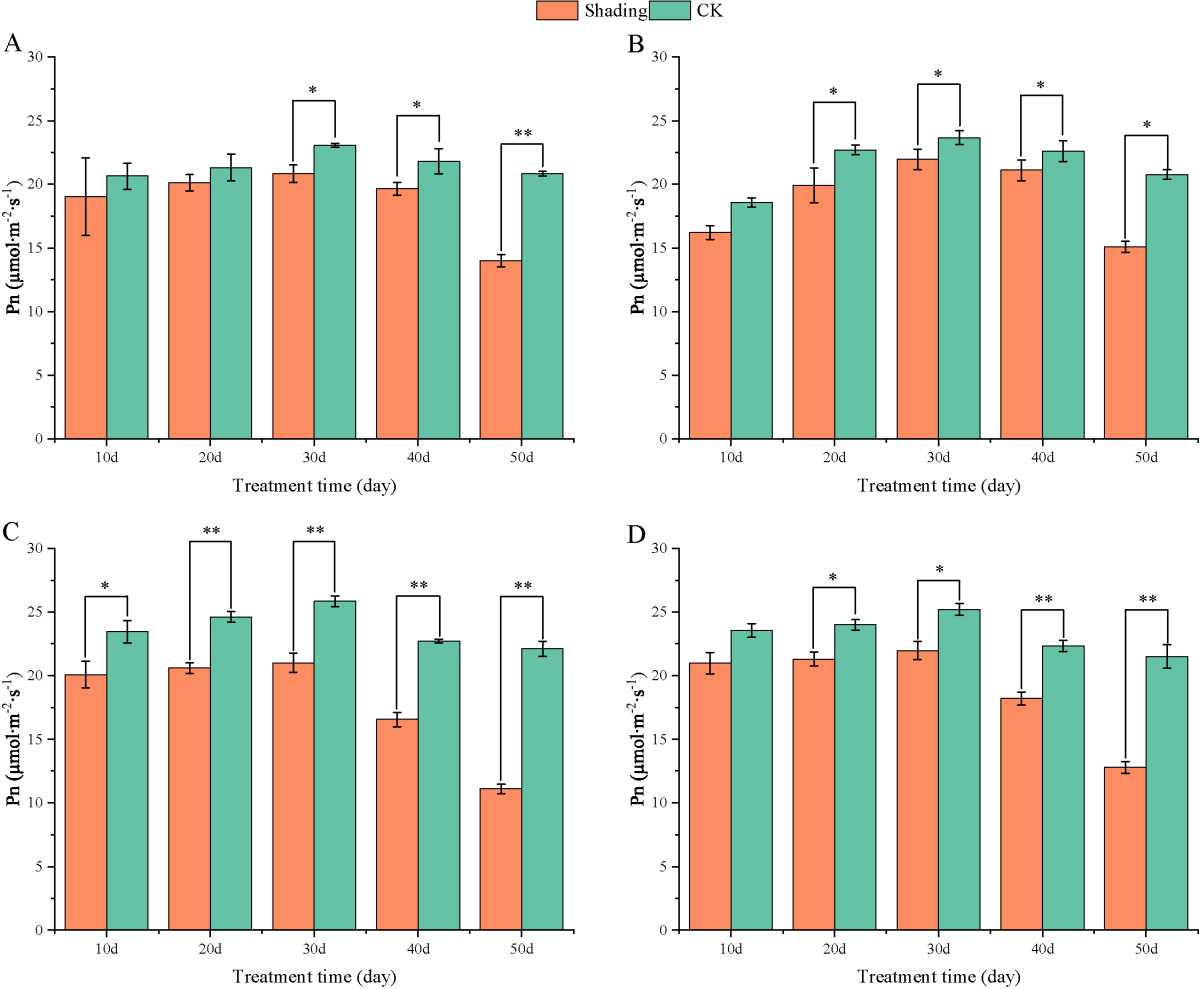
Effect of shading on net photosynthesis rates (P_n_) of peanut leaves at the flowering needle stage (2021). **(A)**, HY22; **(B)**, FH12; **(C)**, NH11; **(D)**, NH5. Y-axis represents net photosynthesis rates (Pn), x-axis represents the days of different cultivars under shade stress. Values are mean of three replicates, bars indicate ± standard error, and stars indicate significant differences between shade and full sun (*P<0.05, **P<0.01).

Under shade stress at the flowering stage, the net photosynthetic rates of different shade-tolerant varieties were lower than those of the control, and the net photosynthetic rates of HY22 decreased highly significant only at 50 days of shade, with 32.80% (**Figure 9A**). The net photosynthesis rates (Pn) of NH11 and NH5 were significantly and highly significant different from that of the control at 10–50 days of shade, and the maximum decrease rate was 49.77% and 40.47% at 50 days of shade, respectively, indicating that shade had an effect on the net photosynthetic rate of shade-sensitive peanut varieties (**Figure 9**).

**Figure 10:**
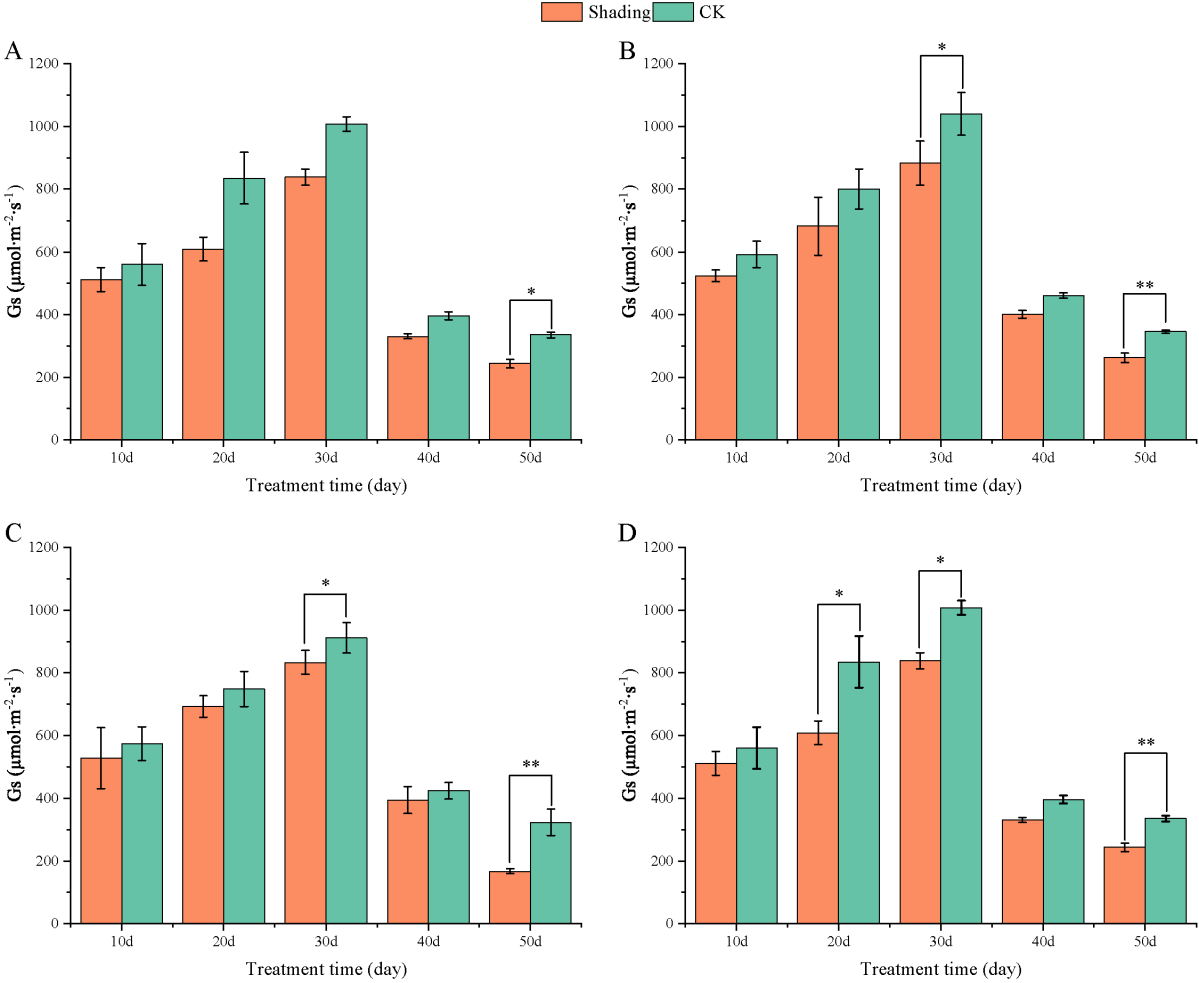
Effect of shading on stomatal conductance (G_S_) of peanut leaves at the flowering needle stage (2021). **(A)**, HY22; **(B)**, FH12; **(C)**, NH11; **(D)**, NH5. Y-axis represents stomatal conductance (G_S_), x-axis represents the days of different cultivars under shade stress. Values are mean of three replicates, bars indicate ± standard error, and stars indicate significant differences between shade and full sun (*P<0.05, **P<0.01).

**Figure 11:**
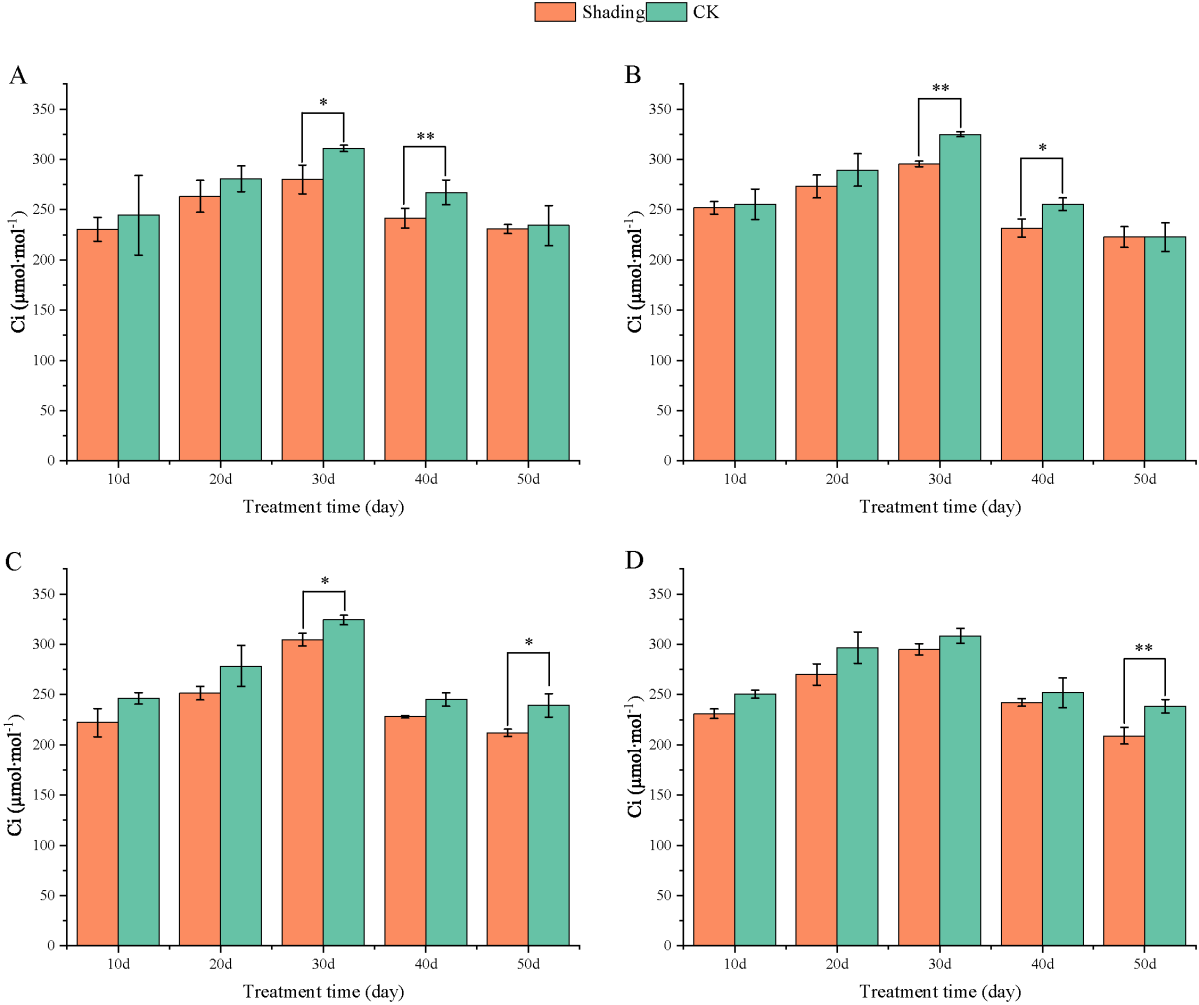
Effect of shading on intercellular CO_2_ concentration (Ci) of peanut leaves at the flowering needle stage (2021). **(A)**, HY22; **(B)**, FH12; **(C)**, NH11; **(D)**, NH5. Y-axis represents intercellular CO_2_ concentration (C_i_), x-axis represents the days of different cultivars under shade stress. Values are mean of three replicates, bars indicate ± standard error, and stars indicate significant differences between shade and full sun (*P<0.05, **P<0.01).

The stomatal conductance (Gs) of peanut leaves decreased compared to that of the control under shade stress at the flowering stage. There was a slight decrease from 10 to 50 days after shade, and the difference was significant in the shade-tolerant peanut varieties HY22 and FH12 at 50 day (**Figure 10**). The Gs of the shade intolerance peanut varieties NH11 had no significant difference compared with that of the control at 10, 20, and 40 days after shade but decreased significant at 30 days while in NH5 reduced significantly at 20 and 30 days. However, it decreased by 24.06% and 27.36% at 50 days after shade, respectively, and the differences were significant (*P*< 0.05) (**Figure 10**). Intercellular CO_2_ concentration (Ci) of peanut leaves decreased compared with that in the control under shade stress at flowering stage. It showed little decrease from 10, 20 and 50 days after shade, and the difference was significant and highly significant at 30 and 40 days in HY22 and FH12. The Ci of NH11 and NH5 were not significantly different from that of the control at 10, 20 and 40 days after shading; however, it decreased by 11.42% and 12.25% 50 days after shading, respectively, with significant and highly significant differences (**Figure 11**).

**Figure 12:**
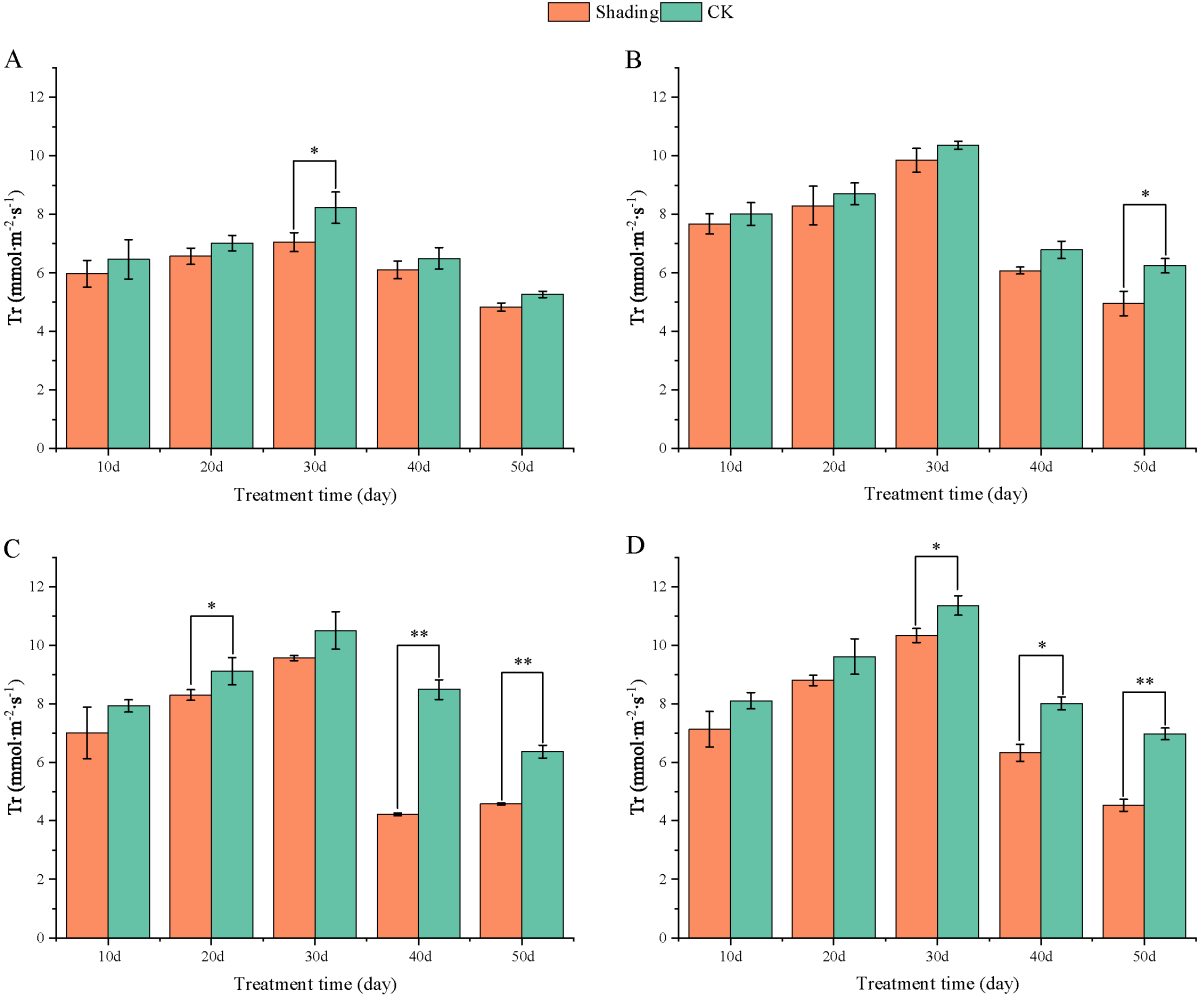
Effect of shading on transpiration rate (T_r_) of peanut leaves at the flowering needle stage (2021). **(A)**, HY22; **(B)**, FH12; **(C)**, NH11; **(D)**, NH5. Y-axis represents transpiration rate (Tr), x-axis represents the days of different cultivars under shade stress. Values are mean of three replicates, bars indicate ± standard error, and stars indicate significant differences between shade and full sun (*P<0.05, **P<0.01).

The transpiration rate (Tr) of peanut leaves decreased compared with that in the control under shade stress at flowering period. The transpiration rate of the leaves of the shade-tolerant peanut variety HY22 showed no significant difference from that of the control at 10, 20, 40 and 50 days after shade. The transpiration rate of the leaves of FH12 showed no significant difference from that of the control at 10–40 days after shade; however, it decreased by 20.65% at 50 days after shade, with significant difference (*P*< 0.05) (**Figure 12**). The transpiration rate of the leaves of NH11 and NH5 were not significantly different from that of the control at 10, 30 and 10, 20 days after shading; however, it decreased by 50.25% and 28.08% and 20.93% and 35.01% at 40 and 50 days after shading, respectively, with significant and highly significant differences (**Figure 12**).

#### 3.4.2 Enzyme activity related to photosynthesis

**Figure 13:**
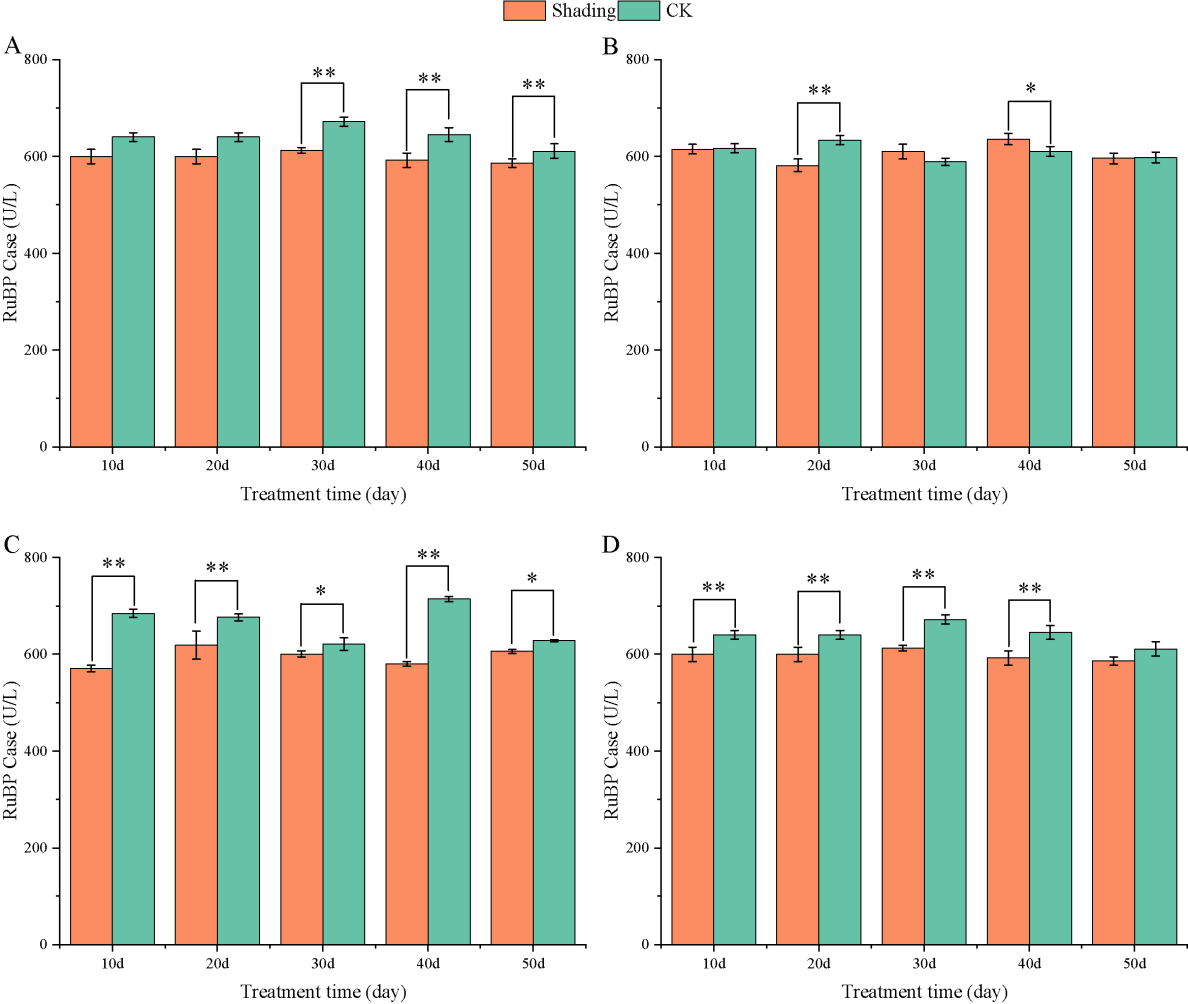
Effect of shading on RuBP Case activity in peanut leaves at the flowering needle stage (2021). **(A)**, HY22; **(B)**, FH12; **(C)**, NH11; **(D)**, NH5. Y-axis represents RuBP Case activity, x-axis represents the days of different cultivars under shade stress. Values are mean of three replicates, bars indicate ± standard error, and stars indicate significant differences between shade and full sun (*P<0.05, **P<0.01).

RuBP carboxylase is a key enzyme in photosynthesis, and its activity has a significant influence on the photosynthetic carbon assimilation ability. The activity of RuBP carboxylase in peanut leaves did not change significantly with the growth process. Compared with that in the control, the RuBP carboxylase activity of different shade-tolerant peanut varieties decreased after shade stress at the flowering stage; however, the difference was highly significant at 30–50 days in HY22 and 20 days in FH12 after shade. Furthermore, the difference between the shade-sensitive peanut varieties and the control group decreased highly significant at 10-40 days after shade (**Figure 13**). The maximum decreases were 10.60% and 9.45% at 20 days in NH11 and NH5.

**Figure 14:**
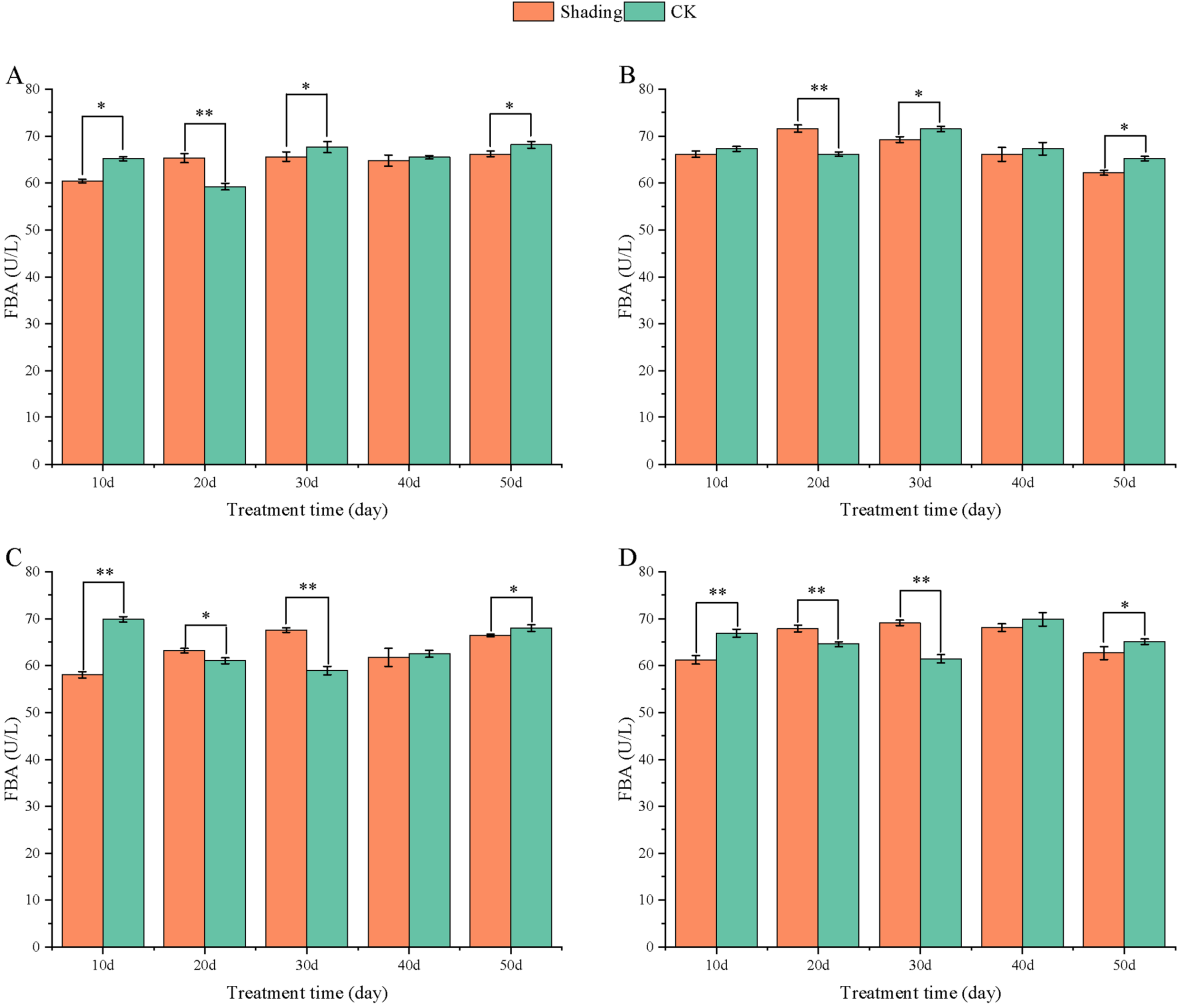
Effect of shading on FBA activity of peanut leaves at the flowering needle stage (2021). **(A)**, HY22; **(B)**, FH12; **(C)**, NH11; **(D)**, NH5. Y-axis represents FBA activity, x-axis represents the days of different cultivars under shade stress. Values are mean of three replicates, bars indicate ± stander error, and stars indicate significant differences between shade and full sun (*P<0.05, **P<0.01).

Compared with that in the control, FBA activity in HY22 and FH12 decreased by 7.29% and 1.65%, respectively, during 10 days of shade stress, while it highly significant decreased in NH11 and NH5 by 16.98% and 8.59%, respectively (**Figure 14**); the difference was significant (*P*< 0.05). The FBPA activity of the shade-tolerant peanut varieties HY22 and FH12 highly significant increased by 10.29% and 8.25% at 20 days after shade stress, while that in the shade-sensitive peanut varieties NH11 and NH5 highly significant increased by 14.63% and 12.35% at 30 days, respectively (**Figure 14**).

### 3.5 Effect of shade on the ultramicroscopic structure of the chloroplasts of peanut leaves at the flowering needle stage

**Figure 15:**
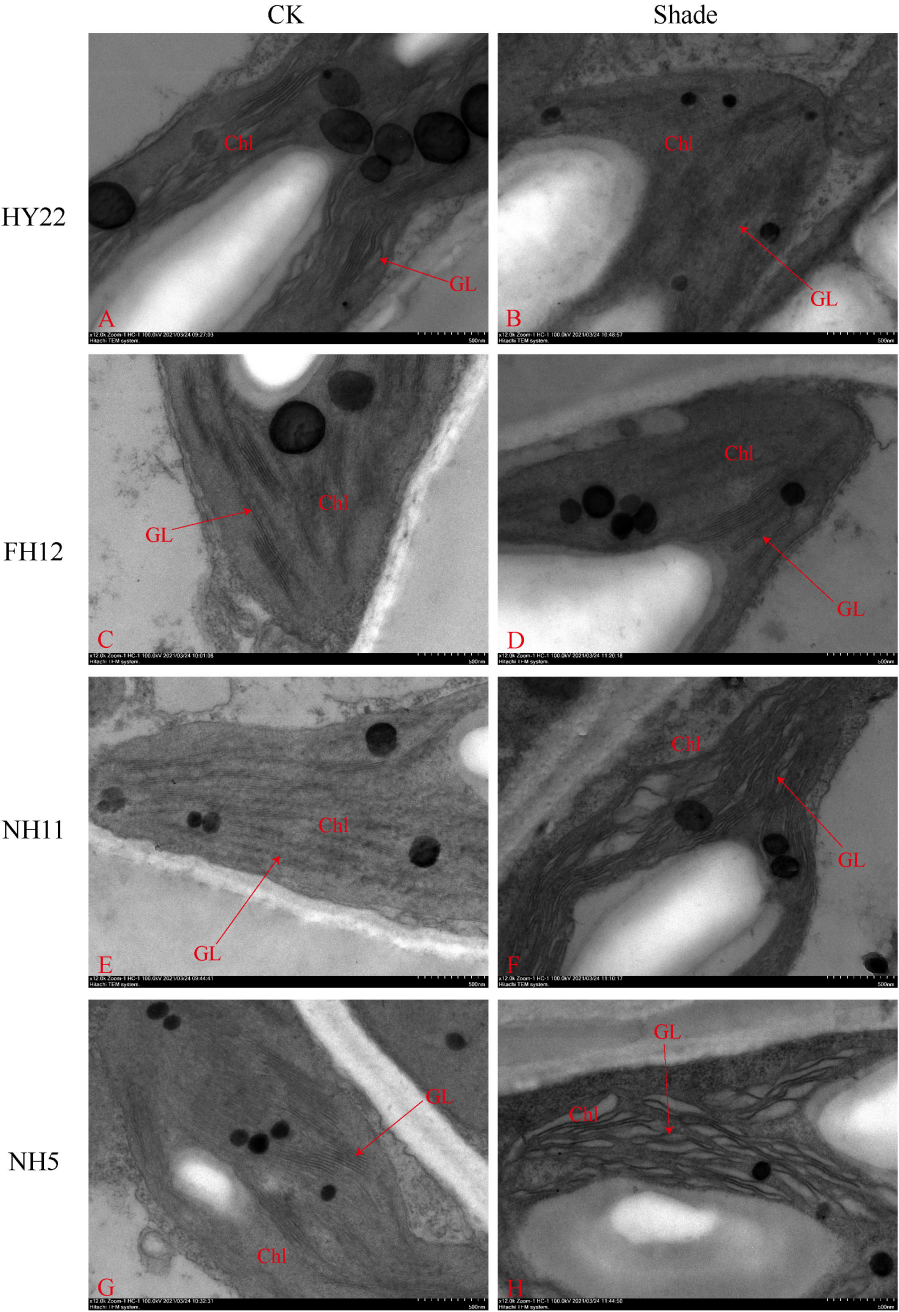
Effect of shading on chloroplast ultrastructure of peanut leaves at the flowering needle stage (2020). **(A)**, HY22 CK. **(B)**, HY22 shade. **(C)**, FH12 CK. **(D)**, FH12 shade. **(E)**, NH11 CK. **(F)**, NH11 shade. **(G)**, NH5 CK. **(H)**, NH5 shade. Chl, chloroplast; GL, basal lamina. Observed at 5.0 k.

**Figure 16:**
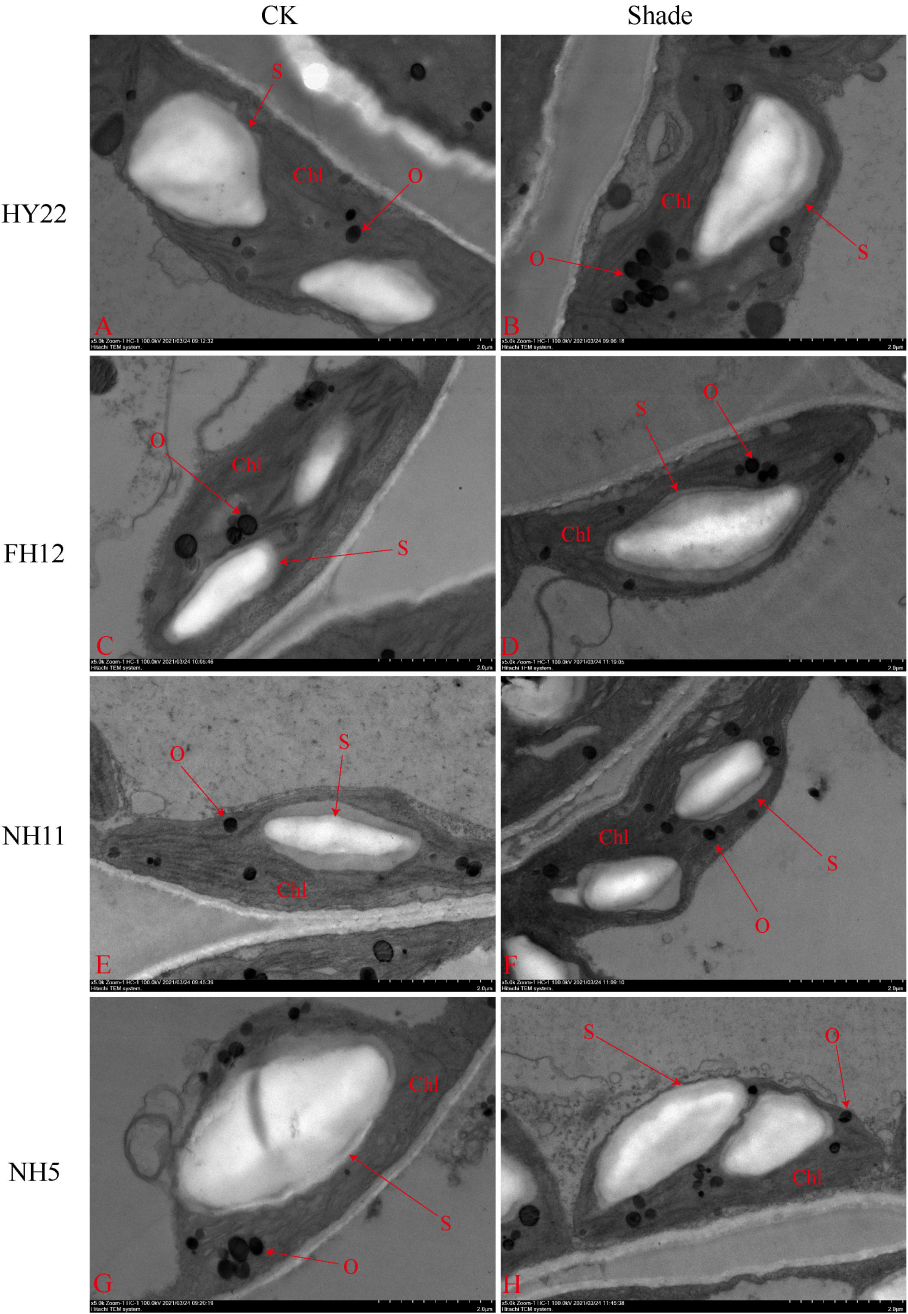
Effect of shading on starch granules ultrastructure of peanut leaves at the flowering needle stage (2020). **(A)**, HY22 CK. **(B)**, HY22 shade. **(C)**, FH12 CK. **(D)**, FH12 shade. **(E)**, NH11 CK. **(F)**, NH11 shade. **(G)**, NH5 CK. **(H)**, NH5 shade. Chl, chloroplast; O, osmiophilic particles; S, starch granules. Observed at 5.0 k.

The ultra-microscopic structure of peanut leaves at the flowering stage changed after shading. Compared with that in the control, the chloroplasts of the shade-tolerant peanut varieties HY22 and FH12 were well developed, and the grana lamella were significantly increased and tightly laminated (**Figures 15**, **16**). However, the grana lamella of NH11 and NH5 were reduced, showing signs of breakage and blurring (**Figures 15**, **16**). The number of starch grains in the leaves of the shade-tolerant peanut varieties decreased, while the volume of starch grains increased. Conversely, although the number of starch grains in the leaves of the shade-sensitive peanut varieties increased, the volume of starch grains decreased. The number of thylakoid particles in leaves of different shade-tolerant peanut varieties increased; however, the volume decreased. The influence of shade stress on the chloroplast ultra-microstructure of the shade tolerant peanut varieties was less than that of shade-intolerant varieties (**Figures 15**, **16**).

### 3.6 The effect of shade on peanuts at the flowering needle stage

Shade has also effect on peanut yield and yield-related indicators at flowering needle stage. The number of fruit branches per plant, pod number per plant, full fruit number per plant, pod weight per plant, 100 fruit weight per plant and 100 kernel weight of peanut varieties with different shade tolerance decreased compared with that in the control; however, the shade-sensitive peanut varieties were more affected than the shade-tolerant varieties by shade (**Supplementary Table S1**). Compared with that in the control, the number of full fruit per plant, 100 fruit weight, and 100 kernel weights of shade-tolerant peanut varieties decreased significantly (*P<* 0.05). Noticeably, those three parameters and the number of fruit branches per plant, pod number per plant, pod weight per plant and the kernel yield of shade-sensitive peanut varieties decreased, and the difference was significant (*P<* 0.05). Changes in the yield components directly affect the yield. Under shade stress at the flowering stage, the yield of different shade-tolerant peanut varieties decreased significantly compared with that in the control. In 2020 and 2021, the yield of HY22 and FH12 decreased by 11.19%, 19.79% and 16.26%, 17.81%, respectively, while that of NH11 and NH5 decreased by 36.01%, 30.07% and 36.67%, 27.14%, respectively. These results indicated that the shade-tolerant peanut varieties were less affected than the shade-intolerant varieties by shade stress.

What’s more, correlation was conducted on chlorophyll a, chlorophyll b, chlorophyll a+b, chlorophyll a/b, and yield (**Table 5**). The results showed that the chlorophyll a/b of different peanut varieties was positively correlated with yield under shading conditions at flowering stage, and the correlation coefficient was 0.618, indicating that the chlorophyll a/b value could be used as one indicator to define peanut yield under shading stress.

**Table 5:** Correlation between shading on chlorophyll and yield in peanut at the flowering needle stage (2021).

	chlorophyll a	chlorophyll b	chlorophyll a+b	chlorophyll a/b	yield
chlorophyll a	1	0.920**	0.994**	-0.224	-0.305
chlorophyll b	0.920**	1	0.957**	-0.581**	-0.491**
chlorophyll a+b	0.994**	0.957**	1	-0.325*	-0.361*
chlorophyll a/b	-0.224	-0.581**	-0.325*	1	0.618**
yield	-0.305	-0.491**	-0.361*	0.618**	1

“**” represent the significant difference at 1% probability level between different treatments. “*”in the table represent the significant difference at 5% probability level between different treatments.

### 3.7 Effect of shade on the quality of peanut kernel at the flowering needle stage

Shade also affects the quality of seed at maturity stage. Compared to that in the control, protein content in seed kernels of different shade-tolerant peanut varieties increased after shade stress at flowering stage, and the differences between NH11 in 2020 and NH5 in 2021 were significant (*P*< 0.05), increasing by 4.03% and 6.63%, respectively (**Supplementary Table S2**). There were no significant differences observed in HY22 and FH12. Regarding the amino acid components, compared with that in the control, the contents of amino acids (except for cysteine) increased, and the increase in the contents of alanine, arginine, aspartic acid, glutamic acid, glycine, histidine, and isoleucine were significant (*P*< 0.05) in NH11 in 2020 and NH5 in 2021 (**Supplementary Table S1**).

Compared with that of the control, the fat content of different shade-tolerant peanut varieties decreased in 2020 after shade stress at the flowering stage. The fat content of the shade-tolerant peanut varieties HY22 and FH12 decreased by 8.23% and 6.31%, respectively, and that of the shade-sensitive peanut varieties NH11 and NH5 decreased by 7.06% and 7.94%, respectively (**Supplementary Table S3**). All the differences were significant (*P*< 0.05); however, the differences in 2021 were not significant, which may be related to the field-test conditions. In 2020 and 2021, the oleic acid content of NH11 and NH5 decreased by 14.05% and 12.50% and 5.95% and 5.19% compared with that in the control, respectively, and the differences were significant (*P*< 0.05). There were no significant differences in the other components compared with that in the control.

## 4 Discussion

### 4.1 Effect of shade on the morphological characteristics of peanut

Light is necessary for crop growth and plays an important role in the growth, development, and morphology of crops. Shade has a significant effect on the growth and development of peanuts, and morphological indices are important factors directly affecting yield formation. In the intercropping system between peanut and maize, the higher the degree of shade received by peanut, the more vigorous the vegetative growth of the plant in the early growth period. The growth parameters include an increase in the length of the main stem, shortening of lateral branches, reduction of branch number, and decrease in dry-matter accumulation, especially at later stage of growth (Yang et al., 2015). However, the influence of shade on leaf area varies. Zhang et al. (2007) demonstrated that soybean leaves in a low-light environment for extended periods were large and thin, with small specific leaf weight, along with soft and long petioles. Moreover, the leaf area of winter wheat increased with the decrease of light intensity, which confirmed that different shade conditions had different effects on the morphological growth of winter wheat (Shi et al., 2019).

Under low-light conditions, peanuts grew taller and thinner with significantly reduced leaf area and specific leaf weight (Rao and Zhao, 1989). Similarly, shade resulted in an increase in plant height and thinning of stems, along with a decrease in the number of main stem branches and leaf area of mung beans (Gong et al., 2022). In this study, the elongation of the main stem, shortening of the lateral branches, and reduction in the leaf area were observed in both shade-tolerant and shade in-tolerant peanut varieties under shade stress compared with those in the control. However, these changes were more evident in shade-sensitive than in shade-tolerant peanut varieties, with significant differences compared with those in the control (**Figures 1**–**6**). Under changing light conditions, crops adjust to the new environment by altering their own morphological traits to chase the light, which is essential for survival. The extension of the main stem is conducive to the plants obtaining more light; however, the increase in the height of the main stem leads to more dry-matter transfer to the stem, which is not conducive to yield formation (Lu et al., 2023). To compete for limited light resources, the lateral growth of stems is inhibited, while the elongation of stems is promoted, and the proportion of photosynthetic products transferred to stems is increased to maintain the availability of nutrients required for their growth. The main stem of shade-tolerant peanut varieties remained low, which was also an important reason for shade tolerance. Furthermore, the larger leaf area of shade-tolerant peanut varieties facilitates higher light-energy interception power and photosynthetic efficiency under shade conditions, with little influence on material production (**Figures 5**, **6**).

Peng and Chen (1999) found that *Arachis pintoi* with robust growth under shade had significantly large leaf area under different levels of shade. In addition, under light and moderate shade stress, the leaf area of soybean with tolerance to shade had relatively sustained (Tan, 2018). Thus, it appeared that large leaf area was an important index for identifying shade-tolerant varieties, laying a solid foundation for capturing more light for photosynthesis and accumulating more carbohydrates. Additionally, the lateral branches of peanuts play a vital role in determining yield as they are the main fruit-bearing branches. The significant decrease in lateral branch length of shade-sensitive peanut varieties while a slight decrease in shade-tolerant peanut varieties (**Figures 3**, **4**), confirmed that shade-tolerant cultivars had the ability to sustain longer lateral branch under shade stress than shade-intolerant cultivars. Taken together, under shade stress, the shade-tolerant cultivars exhibited a more stable morphology than the shade-intolerant cultivars.

### 4.2 Effect of shade on the photosynthesis of peanut

Light is one of the most important ecological factors in plant growth and development. Different light intensities have regulated growth, photosynthesis, morphogenesis, and metabolism of leaf (Shi et al., 2008). Chlorophyll is responsible for the absorption, transmission and transformation of light energy in the plant body. With the weakening of light intensity, the contents of chlorophyll a and chlorophyll b both increased, indicating that soybean seedlings enhanced the photosynthetic intensity by increasing the chlorophyll content to adapt to the weak-light environment (Wu et al., 2014a). Increasing chlorophyll content, particularly chlorophyll b, after shading helps absorb short-wave light, capture light-energy, and improve light-energy utilization efficiency under low-light conditions. Moreover, the contents of chlorophyll a, chlorophyll b, and chlorophyll a+b in peanut leaves increased after shade stress (**Tables 1**, **2**), which confirmed the plants ability to adapt to shade stress, similar to the findings of Lu et al. (2023).

Moreover, Zhang et al. (2010) found that shading not only significantly decreased the chlorophyll content per unit mass of peanut leaves at seedling stage but also significantly reduced the net photosynthetic rate, stomatal conductivity, intercellular CO_2_ concentration, and the activities of RuBP carboxylase and PEP carboxylase and that the degree of influence increased with the degree of shading. Similarly, Pn, Gs, Ci, and Tr were decreased, and significantly reduced in shade-sensitive cultivars (**Figures 9**–**12**). Under shade conditions, Pn of peanut decreased gradually under low-light conditions. Stomatal factors primarily caused Pn reduction, with concurrent deceases in Gs and Ci (Sharkey, 1984). Additionally, nonstomatal factors, such as decreased carboxylation efficiency, led to reduced photosynthetic activity of mesophyll cells (Zhang et al., 2010).

The activity of RuBP carboxylase is closely related to the photochemical efficiency of PS II reaction center, directly impacting Pn. The activities of RuBP carboxylase decreased significantly in shade-sensitive cultivars under shade stress (**Figure 13**), confirming that the shade-sensitive variety was incapable of sustaining normal photosynthesis. The same trend was found in mung bean leaves under shade conditions, such as decreased RuBP carboxylase activity, along with reduced Pn, Tr, and Ci (Gong et al., 2022). Although RuBP carboxylase of the carboxylation stage marks the beginning of the Calvin cycle, the RuBisco-reduction stage is vital to define whether there is sufficient ribulose 5-phosphate for RuBP carboxylase (Cai, 2017). It is of next step that investigate the enzyme changes relating to RuBP reduction. FBA, as an important rate-limiting enzyme in photosynthesis, is the first enzyme to catalyze the conversion of a 3C compound to a 6C compound, that is, to catalyze the reversible synthesis of FBA with dihydroxyacetone phosphate and glyceraldehyde 3-phosphate, which is an essential enzyme involved in starch metabolism, and synthesis of the ^7^C sugar setiheptulose-1, 7-biphosphate (Iwaki et al., 1991; Sonnewald et al., 1994; Flechnera et al., 1999; Wei et al., 2006). FBA plays a key role in the regulation of plant abiotic stress resistance such as low temperature, high temperature, salt stress, drought, strong light. Zhang et al. (2002) found that accumulation of the *FBPA* gene transcripts was increased in the leaf and stem tissues under drought, saline, and Abscisic Acid (ABA) treatment. It was evident that under shade stress, FBA level initially decreased at 10 days and subsequently increased significantly after 20 and 30 days in shade-tolerant and shade-sensitive cultivars, respectively (**Figure 14**). This finding implied that shade stress also had promoted FBA activity, which accumulated faster in shade-tolerant than in shade-sensitive cultivars. The heterologous *FBA* gene can improve the synthesis ability of sugar and starch, increase the photosynthetic efficiency, and significantly enhance the resistance of transgenic plants (Kang et al., 2004; Chen et al., 2006; Ma et al., 2008). Shading can reduce the activities of RuBP carboxylase and *FBPA* in leaves, which further leads to a decrease in the net photosynthetic efficiency.

Photosynthetic capacity of plant organs is reflected by their photosynthetic rate (Feng et al., 2019b). In this study, compared with that in the control, Pn, Gs, Ci, and Tr were decreased in all varieties, with more pronounced reduction in the shade-sensitive cultivars (**Figures 9**, –**12**). The decrease in Pn under shading conditions is primarily caused by the weakening of the light intensity, which was significantly decreased in shade-sensitive varieties after 30–50 days of shading, while it was significantly reduced after 50 days of shading in shade-tolerant cultivars. Stomatal factor is responsible for the decrease in the photosynthetic rate with decreasing Ci and increasing stomatal limit value. Gs and Ci were both decreased in all cultivars but reduced significantly after 50 days of shading in shade-sensitive varieties (**Figures 10**, **11**). However, non-stomatal factor is another reason that the photosynthetic activity of mesophyll cells decreases, RuBP carboxylase efficiency significantly decreased in shade-sensitive varieties but not significantly in shade-tolerant cultivars (**Figure 13**), implying that under the shading treatment, RuBPcase activity was a limiting factor for Pn and that the shading conditions may damage the function of RuBPcase.

### 4.3 Effect of shade on the ultra-microstructure of peanut cells

Shading not only reduces the photosynthetic production capacity but also affects the structure of the photosynthetic organs (Skene, 1974; Ivanova et al., 2008; Huang et al., 2011; Yamazaki and Shinomiya, 2013). Plants living in low-light conditions for extended periods morphologically adjust their leaves based on light intensity and even undergo structural changes to enhance the absorption and utilization ability of low light (Lichtenthaler et al., 1981; Yao et al., 2007). The effects of low light on the chloroplast structure vary depending on the variety and genotype of the crops (Königer and Bollinger, 2012). Wu et al. (2014b) showed that under moderate low-light stress, peanut exhibited an improved ability to capture and use light energy by increasing the light-receiving area of the chloroplast, number of grana, and number of grana lamellae; however, under severe low-light stress, the chloroplast grana development was incomplete, the grana lamellae damaged, and the ability to capture light energy reduced. Under the condition of insufficient light energy, the chloroplast volume of the shade-tolerant peanut varieties increased to obtain enough light for growth (**Figures 15**, **16**). Furthermore, increased granula lamellae, orderly stacking, and clear structure, along with only a few chloroplasts containing starch granules, was observed in tolerant cultivars of tomato (Meng et al., 2021). Similarly, fewer starch granules were present in the shade-tolerant than in the shade-intolerant cultivars (Wu et al., 2014b). However, there were more starch granules in the shade-tolerant cultivars with blurred granula lamellae than that in shade-sensitive cultivars (**Figures 15**, **16**). Thus, the significant decrease of Pn possibly affected the supply of inorganic phosphorus, hindered the transport of sucrose inside and outside the cytoplasm (**Figure 9**), and reduced the release of inorganic phosphorus. Furthermore, triose phosphate was mainly used for starch storage in the chloroplast. The accumulation of a large number of starch granules would cause mechanical damage to the thylakoids and adsorb some carbon assimilation-related enzymes, reducing their enzyme activity (Yao et al., 2007; Meng et al., 2021). Taken together, the shade-tolerant peanut varieties utilize light energy much higher than the shade-intolerant varieties due their improved environmental adaptability; this improved adaptability is mediated by decreased starch grains, intact chloroplast grana layers structure, and increased chloroplast grana layers, which help accelerate the transfer of light energy on thylakoids and improve the ability of peanut to capture light energy (Wu et al., 2014b).

### 4.4 Effects of shade on dry-matter accumulation, yield, and quality of peanut

Yield is a measure of dry-matter accumulation, and a higher amount of dry-matter accumulation is the basis of yield formation, which also depends on the characteristics of dry-matter accumulation and distribution in various organs. Promoting the transfer of dry matter to seeds and increasing the transfer volume of dry matter is the fundamental way to increase yield (Wang et al., 2021). Shade affects the accumulation and transport of dry matter in crops; it restricts photosynthesis, resulting in a significant decrease in total dry-matter mass under shade stress (Zhang et al., 2018). Moreover, Cai et al. (2009) found that the accumulation of dry matter in different aboveground organs was inhibited during pre-flowering shading of a strong gluten wheat variety. In this study, the dry-matter accumulation in the entire plant and organs of different shade-tolerant peanut varieties under shade decreased compared with that in the control; however, the rate of decline in shade-sensitive peanut varieties was greater than that in the shade-tolerant varieties, and reached a significant level during the late shade period (**Figures 7**, **8**). These results indicate that the shade-tolerant peanut varieties maintained relative stable chloroplast structure, accelerated the transfer of light energy, and improved nutrient transport compared with that by the shade-sensitive peanut varieties, which transferred more nutrients to the grain and adapted to the shade environment.

Leaf photosynthesis is the foundation of crop grain formation, and yield components directly affect yield formation (Liu et al. 2015). Fan et al. (2016) found that low light reduced the yield per plant by reducing pod number per plant, kernel number per plant, and 100-kernel weights. Under shade conditions, the seed setting rate, 1,000-grain weight, and yield of rice decreased significantly (Pan et al., 2016), while it was more obvious in shade-sensitive rice cultivars (Chen et al., 2019b; Du et al., 2014). Similarly, in this study, shade significantly reduced the number of full fruits per plant, 100-fruit weight, and 100-kernel weights of peanuts, thereby significantly reducing peanut pod yield (**Supplementary Tables S1–S3**). Insufficient light intensity directly affected the peanut photosynthesis and dry-matter accumulation. Furthermore, the dysfunctional development of lateral branches could also account for the decrease of pod yield due to lateral branches being the main fruiting branches (Lin et al., 2020). Compared with the shade-sensitive peanut varieties, shade-tolerant peanut varieties maintained the morphology and physiology, and the rate of full fruit number per plant, 100-fruit weight, and 100-kernel weight decreased slightly, which gave solid foundation for the yield, and shade tolerance.

Studies have shown that the change in light conditions affects the physiological processes such as photosynthesis, nutrient absorption, and redistribution in the plant and ultimately affect the formation of crop quality. Ren et al. (2003) found that under low-light stress, poor grain filling led to poor rice-milling and appearance. However, Campbell et al. (1987) demonstrated that the reduction in light intensity increased the crude protein content in the grains. Furthermore, the protein content of peanut kernel increased, while the fat content was less affected under the moderate and mild drought stress at the seedling stage. Under severe drought conditions, the fat content of peanut kernel significantly reduced, while the protein content was only slightly affected (Yan et al., 2007). Han et al. (2014) found that the protein content of peanut kernel increased and the fat content decreased after intercropping with shading stress. Herein, peanut kernel protein content increased and fat content decreased under shade stress. Thus, the decrease in the photosynthetic capacity and dry-matter accumulation in each organ and whole plant was harmful to the formation of yield, and the significant decrease of number of full fruits per plant, 100-fruit weight, and 100-kernel weight directly led to the decrease of yield (**Supplementary Tables S1–S3**). However, shade-tolerant varieties in this study maintained relative better morphological and physiological level to obtain higher yield than shade-intolerant varieties.

## 5 Conclusion

The shade-tolerant peanut varieties exhibited significant differences in their tolerance to the shade than the shade-intolerant varieties. Shade-tolerant peanut varieties showed relatively shorter main stem height and longer lateral branch than the control; however, the difference was not significant. The leaf area and dry-matter accumulation of shade-tolerant peanut varieties were significantly increased during the late stage of shade stress. Although Gs and Ci decreased due to the insufficient light energy, the increased chloroplast grana layers, intact chloroplast grana layers structure, increased starch grains volume, less decreased RuBP enzyme and early functioned FBA enzyme had maintained Pn thereby accelerating the transfer of light energy to the thylakoids. Consequently, in 2020 and 2021, the yield of the shade-tolerant peanut varieties HY22 and FH12 decreased by 11.19%, 19.79%, and 16.26%, 17.81%, respectively, while that of the shade-sensitive peanut varieties NH11 and NH5 decreased by 36.01%, 30.07% and 36.67%, 27.14%, respectively.

## Data Availability

The raw data supporting the conclusions of this article will be made available by the authors, without undue reservation.
