# Supplementary material for: Effects of shading on morphology, photosynthesis characteristics, and yield of different shade-tolerant peanut varieties at the flowering stage
Source: Front Plant Sci. 2024 Oct 8;15:1429800. doi: 10.3389/fpls.2024.1429800 (PMC11493661; doi:10.3389/fpls.2024.1429800)
Supplement: Supplementary file 1 [file Table1.docx]

Supplementary Table 1 Effects of shading on peanut yield and related agronomic characters at flowering needle stage

| Year | Cultivar | Treatment | Number of fruiting branches per plant | Number of pods per plant | Number of full pods per plan | Pods weight per plant/g | Full pods weight per plant/g | 100-Pods weight/g | 100-kernel weight/g | Seed kernel yield | Yield/kg·hm^-2^ |
| --- | --- | --- | --- | --- | --- | --- | --- | --- | --- | --- | --- |
| 2020 | HY22 | CK | 7.45±0.93a | 20.63±2.25a | 16.63±1.13a | 22.67±1.08a | 22.17±2.19a | 150.60±2.98a | 122.15±0.59a | 0.68±0.01a | 4612.07±287.93a |
|  |  | Shade | 6.64±1.12a | 16.00±1.07a | 13.25±1.06b | 18.71±1.20a | 19.70±1.88a | 130.87±1.29b | 95.35±1.58b | 0.67±0.02a | 4095.91±192.50b |
|  | FH12 | CK | 7.07±1.60a | 19.21±1.62a | 16.00±1.64a | 20.39±1.47a | 18.14±1.42a | 136.06±0.89a | 96.21±1.31a | 0.74±0.01a | 4482.76±318.91a |
|  |  | Shade | 7.13±1.17a | 16.43±0.71a | 12.86±1.02b | 17.52±1.10a | 15.69±1.24a | 111.20±0.64b | 75.46±2.16b | 0.73±0.01a | 3595.45±138.23b |
|  | NH11 | CK | 7.93±2.16a | 23.13±1.26a | 18.80±1.18a | 23.72±1.63a | 22.14±1.71a | 140.75±1.72a | 106.57±1.31a | 0.68±0.01a | 4683.91±191.43a |
|  |  | Shade | 6.78±1.41b | 12.47±0.60b | 10.33±0.56b | 12.13±0.82b | 11.24±0.82b | 107.21±0.86b | 83.23±2.16a | 0.73±0.01b | 2997.37±183.32b |
|  | NH5 | CK | 9.25±1.47a | 24.29±1.79a | 17.50±1.94a | 26.98±3.35a | 23.46±3.45a | 152.14±0.56a | 104.15±0.47a | 0.73±0.01a | 4137.93±175.95a |
|  |  | Shade | 6.99±1.96b | 16.36±1.20b | 10.93±1.12b | 14.32±1.09b | 12.25±1.24b | 102.50±1.21b | 79.50±1.28b | 0.76±0.01b | 2893.68±117.86b |
| 2021 | HY22 | CK | 8.29±0.82a | 24.00±1.07a | 17.75±1.03a | 23.42±1.07a | 19.91±1.75a | 138.71±1.68a | 96.18±2.01a | 0.63±0.01a | 4448.27±56.31a |
|  |  | Shade | 8.20±1.01a | 20.60±1.23a | 13.40±0.74b | 21.87±1.32a | 17.01±2.22a | 113.11±1.32b | 75.64±1.43b | 0.62±0.01a | 3725.03±189.45b |
|  | FH 12 | CK | 7.00±1.01a | 18.83±1.21a | 14.79±1.32a | 20.36±1.22a | 18.41±1.33a | 122.19±0.99a | 107.47±2.31a | 0.63±0.01a | 4155.26±289.21a |
|  |  | Shade | 6.80±1.32a | 16.33±1.16a | 11.10±0.38b | 17.50±1.16a | 14.91±1.26a | 96.43±1.10b | 95.53±1.56b | 0.63±0.01a | 3415.17±156.89b |
|  | NH 11 | CK | 8.88±1.43a | 26.88±0.86a | 17.38±1.17a | 23.90±1.11a | 19.77±1.21a | 133.74±1.03a | 113.52±1.23a | 0.63±0.01a | 4456.45±146.83a |
|  |  | Shade | 7.00±1.63b | 16.29±0.78b | 7.71±0.68b | 11.51±0.84b | 7.94±0.68b | 97.66±0.69b | 81.65±1.02b | 0.67±0.01b | 2822.12±172.43b |
|  | NH 5 | CK | 8.43±1.25a | 23.11±1.68a | 16.33±1.93a | 24.82±2.15a | 20.72±3.21a | 135.06±1.32a | 99.22±1.31a | 0.66±0.01a | 4298.76±198.27a |
|  |  | Shade | 6.50±1.24b | 14.60±0.98b | 9.00±1.13b | 12.08±1.02b | 9.93±1.23b | 92.60±2.33b | 67.10±1.20b | 0.69±0.01b | 3132.28±235.13b |

Note: Different lowercase letters in the same column in each index indicated significant differences among samples (*P*<0.05), ± indicates standard error.

Supplementary Table 2 Effect of shading on protein and amino acid composition of peanut kernel at flowering needle stage

Unit: %

| Year | Cultivar | Treatment | Pro | ALA | ARG | ASP | Cys | GLY | Gly | HIS | ILE |
| --- | --- | --- | --- | --- | --- | --- | --- | --- | --- | --- | --- |
| 2020 | HY22 | CK | 23.62±0.44a | 0.94±0.01a | 2.95±0.06a | 2.95±0.06a | 0.16±0.03a | 5.41±0.12a | 1.43±0.01a | 0.59±0.01a | 0.79±0.02a |
|  |  | Shade | 24.79±0.08a | 0.97±0.01a | 3.16±0.02a | 3.14±0.01a | 0.14±0.01a | 5.75±0.03a | 1.48±0.01a | 0.61±0.00a | 0.83±0.01a |
|  | FH12 | CK | 24.66±0.46a | 0.99±0.01a | 3.12±0.07a | 3.09±0.07a | 0.15±0.02a | 5.60±0.13a | 1.48±0.01b | 0.61±0.01a | 0.81±0.02a |
|  |  | Shade | 25.95±0.12a | 1.03±0.01a | 3.35±0.01a | 3.26±0.02a | 0.15±0.01a | 5.92±0.05a | 1.54±0.01a | 0.64±0.00a | 0.83±0.01a |
|  | NH11 | CK | 24.98±0.28a | 0.98±0.01b | 3.14±0.05b | 3.14±0.03b | 0.13±0.03a | 5.75±0.04b | 1.46±0.02b | 0.62±0.01b | 0.85±0.01b |
|  |  | Shade | 26.08±0.14b | 1.01±0.00a | 3.35±0.02a | 3.30±0.01a | 0.13±0.02a | 6.00±0.03a | 1.51±0.01a | 0.65±0.01a | 0.88±0.00a |
|  | NH5 | CK | 23.49±0.35a | 0.93±0.01a | 2.94±0.08a | 2.92±0.05a | 0.17±0.02a | 5.37±0.08a | 1.44±0.01a | 0.58±0.01a | 0.77±0.02a |
|  |  | Shade | 25.34±0.23a | 0.99±0.01a | 3.24±0.03a | 3.21±0.04a | 0.15±0.03a | 5.87±0.10a | 1.52±0.01a | 0.62±0.00a | 0.82±0.00a |
| 2021 | HY22 | CK | 24.26±0.28a | 0.98±0.01a | 3.10±0.05a | 3.05±0.04a | 0.20±0.00a | 5.58±0.07a | 1.45±0.01a | 0.61±0.01a | 0.82±0.01a |
|  |  | Shade | 24.86±0.33a | 1.00±0.01a | 3.19±0.05a | 3.13±0.03a | 0.19±0.00a | 5.75±0.07a | 1.47±0.02a | 0.63±0.01a | 0.83±0.00a |
|  | FH12 | CK | 25.65±0.27a | 1.03±0.01a | 3.28±0.03a | 3.19±0.05a | 0.16±0.01a | 5.86±0.09a | 1.50±0.01a | 0.64±0.00a | 0.83±0.02a |
|  |  | Shade | 26.43±0.71a | 1.06±0.02a | 3.45±0.13a | 3.33±0.10a | 0.15±0.03a | 6.06±0.14a | 1.54±0.03a | 0.66±0.02a | 0.85±0.02a |
|  | NH11 | CK | 25.11±0.26a | 1.02±0.01a | 3.21±0.03a | 3.15±0.02a | 0.17±0.01a | 5.77±0.05a | 1.47±0.02a | 0.63±0.00a | 0.84±0.01a |
|  |  | Shade | 25.22±0.17a | 1.01±0.01a | 3.26±0.04a | 3.19±0.02a | 0.16±0.03a | 5.81±0.02a | 1.47±0.01a | 0.64±0.00a | 0.86±0.01a |
|  | NH5 | CK | 24.28±0.30b | 0.98±0.01b | 3.10±0.05b | 3.01±0.02b | 0.19±0.02a | 5.53±0.03b | 1.46±0.01b | 0.61±0.01b | 0.81±0.00b |
|  |  | Shade | 25.89±0.21a | 1.03±0.01a | 3.40±0.03a | 3.27±0.03a | 0.18±0.02a | 5.97±0.07a | 1.53±0.01a | 0.64±0.00a | 0.84±0.00a |

Note: Different lowercase letters in the same column in each index indicated significant differences among samples (*P*<0.05), ± indicates standard error.

Supplementary Table 3 Effect of shading on fat and fatty acid components of peanut kernel at flowering needle stage

Unit: %

| Year | Cultivar | Treatment | Oil | C16:0 | C18:0 | C18:1 | C18:2 | C18:3 | C20:1 | C22:1 |
| --- | --- | --- | --- | --- | --- | --- | --- | --- | --- | --- |
| 2020 | HY22 | CK | 48.73±0.94a | 11.00±0.04a | 3.35±0.03a | 43.77±0.45a | 34.61±0.49a | 0.05±0.00a | 1.57±0.05a | 1.08±0.21a |
|  |  | Shade | 44.72±0.64b | 11.15±0.03a | 2.75±0.06b | 44.26±0.23a | 34.23±0.51a | 0.04±0.00a | 1.44±0.04a | 0.73±0.05a |
|  | FH12 | CK | 47.07±0.66a | 11.11±0.05a | 3.05±0.17a | 43.94±0.28a | 33.64±0.07a | 0.05±0.00a | 1.59±0.08a | 0.90±0.37a |
|  |  | Shade | 44.10±0.49b | 11.29±0.05a | 2.71±0.08a | 43.48±0.17a | 33.72±0.15a | 0.04±0.00a | 1.53±0.05a | 0.80±0.14a |
|  | NH11 | CK | 49.02±0.70a | 10.91±0.11a | 3.56±0.01a | 44.59±0.38a | 33.17±0.28a | 0.05±0.00a | 1.78±0.03a | 1.28±0.05a |
|  |  | Shade | 45.56±0.51b | 11.34±0.13a | 3.03±0.11b | 43.81±0.76a | 34.94±0.57a | 0.04±0.00a | 1.53±0.04b | 1.00±0.18a |
|  | NH5号 | CK | 48.12±0.30a | 11.31±0.00a | 3.03±0.08a | 42.13±0.20a | 36.26±0.26a | 0.05±0.00a | 1.52±0.03a | 1.09±0.17a |
|  |  | Shade | 44.30±0.64b | 11.75±0.35a | 2.55±0.26a | 41.40±0.79a | 37.53±0.56a | 0.04±0.00a | 1.33±0.02b | 0.62±0.24b |
| 2021 | HY22 | CK | 46.74±0.38a | 11.72±0.14a | 3.66±0.13a | 41.85±0.55a | 35.23±0.20a | 0.05±0.00a | 1.60±0.02a | 1.49±0.12a |
|  |  | Shade | 44.80±0.81a | 11.84±0.07a | 3.37±0.19a | 41.58±0.80a | 35.84±0.40a | 0.05±0.00a | 1.56±0.08a | 1.43±0.13a |
|  | FH12 | CK | 46.26±0.23a | 11.77±0.13a | 3.68±0.01a | 42.42±0.66a | 35.50±0.33a | 0.05±0.00a | 1.61±0.04a | 1.43±0.07a |
|  |  | Shade | 44.43±1.09a | 11.89±0.16a | 3.47±0.11a | 41.26±0.74a | 36.81±0.54a | 0.05±0.00a | 1.59±0.02a | 1.13±0.21a |
|  | NH11 | CK | 46.90±0.17a | 11.85±0.02a | 3.88±0.10a | 41.51±0.28a | 35.34±0.61a | 0.05±0.00a | 1.68±0.04a | 1.58±0.02a |
|  |  | Shade | 45.19±0.52a | 12.00±0.22a | 3.46±0.03a | 40.72±0.95a | 35.88±0.75a | 0.05±0.00a | 1.58±0.04b | 1.30±0.22a |
|  | NH5 | CK | 46.52±0.75a | 11.75±0.09b | 3.62±0.06a | 41.34±0.38a | 36.55±0.23b | 0.05±0.00a | 1.54±0.02a | 1.66±0.17a |
|  |  | Shade | 43.84±0.51a | 12.22±0.07a | 3.17±0.12b | 39.44±0.49a | 38.75±0.40a | 0.05±0.00a | 1.46±0.03b | 1.21±0.14a |

Note: Different lowercase letters in the same column in each index indicated significant differences among samples (*P*<0.05), ± indicates standard error.
